# NG-Tax, a highly accurate and validated pipeline for analysis of 16S rRNA amplicons from complex biomes

**DOI:** 10.12688/f1000research.9227.2

**Published:** 2018-11-23

**Authors:** Javier Ramiro-Garcia, Gerben D. A. Hermes, Christos Giatsis, Detmer Sipkema, Erwin G. Zoetendal, Peter J. Schaap, Hauke Smidt

**Affiliations:** 1 TI Food and Nutrition (TIFN), Wageningen, 6703 HB, The Netherlands; 2 Laboratory of Microbiology, Wageningen University, Wageningen, 6708 WE, The Netherlands; 3 Laboratory of Systems and Synthetic Biology, Wageningen University, Wageningen, 6708 WE, The Netherlands; 4 Aquaculture and Fisheries Group, Wageningen University, Wageningen, 6708 WD, The Netherlands

**Keywords:** 16S rRNA amplicon analysis, microbial community analysis, microbial ecology, next-generation sequencing, bioinformatic pipeline

## Abstract

**Background: **Massive high-throughput sequencing of short, hypervariable segments of the 16S ribosomal RNA (rRNA) gene has transformed the methodological landscape describing microbial diversity within and across complex biomes. However, several studies have shown that the methodology rather than the biological variation is responsible for the observed sample composition and distribution. This compromises meta-analyses, although this fact is often disregarded.

**Results: **To facilitate true meta-analysis of microbiome studies, we developed NG-Tax, a pipeline for 16S rRNA gene amplicon sequence analysis that was validated with different mock communities and benchmarked against **QIIME **as a frequently used pipeline. The microbial composition of 49 independently amplified mock samples was characterized by sequencing two variable 16S rRNA gene regions, V4 and V5-V6, in three separate sequencing runs on Illumina’s HiSeq2000 platform. This allowed for the evaluation of important causes of technical bias in taxonomic classification: 1) run-to-run sequencing variation, 2) PCR–error, and 3) region/primer specific amplification bias. Despite the short read length (~140 nt) and all technical biases, the average specificity of the taxonomic assignment for the phylotypes included in the mock communities was 97.78%. On average 99.95% and 88.43% of the reads could be assigned to at least family or genus level, respectively, while assignment to ‘spurious genera’ represented on average only 0.21% of the reads per sample. Analysis of α- and β-diversity confirmed conclusions guided by biology rather than the aforementioned methodological aspects, which was not achieved with QIIME.

**Conclusions: **Different biological outcomes are commonly observed due to 16S rRNA region-specific performance. NG-Tax demonstrated high robustness against choice of region and other technical biases associated with 16S rRNA gene amplicon sequencing studies, diminishing their impact and providing accurate qualitative and quantitative representation of the true sample composition. This will improve comparability between studies and facilitate efforts towards standardization.

## Background

Recent advances in massive high-throughput, short-amplicon sequencing are revolutionizing efforts to describe microbial diversity within and across complex biomes ^1^. Cultivation-independent whole metagenome sequencing has received increasing attention in the functional characterization of individual communities. These efforts, however, remain relatively expensive on a per sample basis, and the richer but much more unstructured information content requires complex data modelling and analysis procedures ^2^. Therefore targeted surveys for specific taxonomic marker genes, such as the 16S ribosomal RNA (rRNA) gene ^3, 4^, remain essential in many microbial ecological studies. These surveys rely on sequencing of short, PCR amplified, hypervariable subregions rather than the full-length gene, mostly for reasons of throughput, sequence depth and cost-efficiency.

Despite great efforts to address the accuracy and reproducibility of scientific insights generated from 16S rRNA gene amplicon sequencing studies, methodology rather than biology has been shown to be the largest driver of variation in many microbiome studies ^5– 13^, hampering comparability. The increased levels of standardization in analysis pipelines have enhanced *replicability* rather than *reproducibility*, by providing widely adopted defaults ^11^. However, there is a large dinstintion between the two. Drummond ^14^ suggested that exact replication of an experiment ( *i.e*., replicability) is less informative (although a necessary pre-requisite for any scientific endeavour) than the corroboration of findings by reproduction in different independent setups ( *i.e*., reproducibility) ^15^, because biological findings that are robust to independent methodologies are arguably more dependable than any single-track analysis ^11^. This distinction is highly relevant for the field of microbial ecology, where replicability is often confused with reproducibility, which is apparent from many often non-interchangeable methodologies.

Accuracy can typically be evaluated by the addition of positive controls. Generally these are synthetic or mock communities (MCs) consisting of phylotypes that, ideally, are representative of the ecosystem of interest. MCs allow researchers to answer two essential questions concerning accuracy. 1) Do I retrieve the number of species I put in, and if so are they correctly assigned? 2) How well does the PCR, sequencing and data analysis procedure reproduce species relative abundances? Reproducibility can be evaluated by comparing separate sequencing runs and different primer pairs that cover distinct 16S rRNA gene regions. Although replicability is often achieved, accuracy has been shown to be challenging especially at higher taxonomic resolution such as at genus level ^16, 17^.

Central to all 16S rRNA gene amplicon studies are Operational Taxonomic Units (OTUs). These are often regarded as a synthetic proxy for microbial species and are typically clustered at 97% sequence similarity. However, the prokaryotic species definition remains a hotly debated topic without any satisfying solution so far ^18– 20^. Moreover, the 97% sequence similarity threshold is based on the complete 16S rRNA gene (~1500 nt), and although sequence variability is not evenly distributed it is routinely applied to short reads of 100–500 nt. Different regions would therefore require their own species level cut-off. The combination of an ambiguous prokaryotic species definition and its application to short reads is the foundation for many complications regarding ‘correct’ OTU clustering. So far, there is little consensus on key experimental choices such as primers, targeted variable regions and OTU picking/clustering algorithms. Each of these technical aspects generate biases, and different methods produce clearly distinct results, leading to a situation where results of current studies cannot be easily compared or extrapolated to other study designs.

Historically, 16S rRNA gene sequences generated in a project were initially clustered *de novo* into OTUs at >97% sequence similarity using various clustering algorithms, mostly because available 16S rRNA gene reference databases were thought to provide insufficient coverage ^21– 24^. Although new clustering algorithms that reduce the influence of clustering parameters, such as a hard cutoff for cluster similarity, have been specifically developed for amplicons ^25^, cluster generation is context-dependent, *i.e*. different datasets generate different clusters, and different algorithms may produce different end-results ^10, 11^. Therefore, even though the same analysis framework is used, independent studies remain incomparable at OTU level. Consequently, reference-based OTU clustering has received increasing attention, due to the need for standardization, and because *de-novo* OTU clustering for very large datasets, such as those generated by Hiseq and Miseq sequencers has become computationally very intensive, unless greedy heuristics are employed which suffer from the problems described above. With reference-based OTU clustering, sequences are mapped to pre-clustered reference sets of curated 16S rRNA gene sequences, provided by dedicated databases such as the Ribosomal Database Project (RDP), Greengenes and SILVA ^26– 28^. The consequence of this approach is that the ‘quality’ of the clustering of the reference set propagates to reference-picked OTUs. Clustering has limited robustness ^10, 11, 29^, and unbalances in databases due to over- or under-representation of certain species as well as error hotspots that are not necessarily matched to the variable regions ^8^, can potentially lead to a biased cluster formation, driven by non-biological factors. These effects have been previously ignored or underestimated in reference OTU picking protocols ^11^.

Another essential experimental choice concerns the selection of a targeted variable region of the 16S rRNA gene, because it should represent the sequence variability encountered with the full-length gene. Despite several studies comparing the performance of diverse regions, sequence lengths, sequencing platforms and taxon assignment methodologies, both within and across laboratories ^5, 6, 8, 30– 33^, there still is no complete consensus about the best variable regions of the 16S rRNA gene to asses, although some initiatives such as the Earth Microbiome Project ^34^ are setting some standards that are increasingly being adopted by the field. There are several factors that can lead to the commonly observed highly region-specific differences across datasets: 1) PCR bias of varying degrees ^6, 8, 35^, 2) different regions are associated with different error profiles and different rates of chimera formation ^8, 36^, and 3) the actual variation contained in the sequence is dissimilar ( *e.g.* some regions are not variable enough to differentiate between genera, while others are), which in turn can affect clustering ^11^.

Apart from the use of a diverse range of primers and OTU picking protocols that can cause differences in results between studies and/or laboratories, sequencing error is a third important factor that defines data quality. Massive high throughput, short read length sequencing platforms have not been developed for amplicon sequencing but rather for whole genome sequencing, where sequence errors in individual reads is less important. However, in 16S rRNA gene amplicon sequencing every sequencing error could potentially lead to an incorrect OTU classification which may ultimately lead to the false discovery of a new phylotype. To avoid overestimation of microbial diversity, stringent quality filtering is therefore considered essential ^16^.

To address all of the aforementioned challenges associated with microbiota profiling, multiple standardized mock communities (MCs) were specifically designed. Those MCs were sequenced in multiple sequencing runs using a Illumina Hiseq2000 instrument (101nt paired end). Furthermore, two tandem variable 16S rRNA gene regions were sequenced in parallel (V4 and V5-V6). This led to the development of NG-Tax, a pipeline that accounts for biases associated with technical aspects associated with 16S rRNA gene amplicon sequencing. Therefore, NG-Tax will improve comparability by removing technical bias and facilitate efforts towards standardization, by focusing on reproducibility as well as accuracy. To assess the performance regarding key output parameters such as taxonomic classification, composition, richness and diversity measures we benchmarked the results obtained with NG-Tax with results obtained with QIIME ^13^, a common pipeline used for the analysis of this type of data.

## Results and discussion

### NG-Tax layout

**Figure 1.**
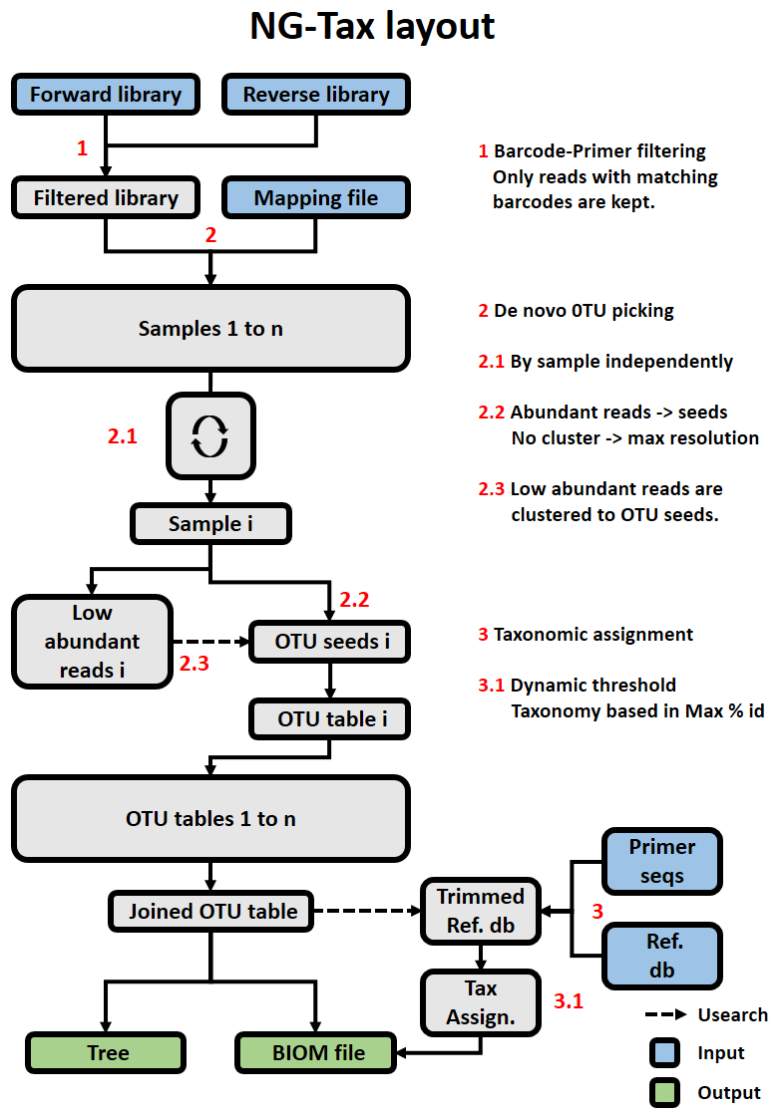
NG-Tax layout. Input files are depicted in blue, output files are depicted in green and clustering processes using usearch are indicated with dashed lines. Details for some steps of the pipeline are marked with red numbers.

NG-Tax consists of three core elements, namely barcode-primer filtering, OTU-picking and taxonomic assignment ( Figure 1). Examples of use and details of each step of the pipeline can be found in the user manual in Dataset 1.

***Barcode-Primer filtering.*** In a first step, paired end libraries are combined, and only read pairs with perfectly matching primers and barcodes are retained. To this end, both primers are barcoded to facilitate identification of chimeras produced during library generation after pooling of individual PCR products.

***OTU picking.*** For each sample an OTU table is created with the most abundant sequences, using a minimum user defined relative abundance threshold. In this particular study we employed a threshold of 0.1% minimum relative abundance. Lowering the threshold will lead to the acceptance of low abundant OTUs, with an increased probability of these OTUs being artifacts due to sequencing and PCR errors. Abundance thresholds are commonly used to remove spurious OTUs generated by sequencing and PCR errors ^17, 37^, but previous studies applied thresholds defined by the complete dataset, thereby ignoring sample size heterogeneity which may lead to under-representation of asymmetrically distributed OTUs.

Commonly employed quality filtering parameters based on Phred score, such as minimum average Phred score, maximum number of ambiguous positions, maximum bad run length, trimming and minimum read length after quality trimming, are not utilized in NG-Tax because quality scores from the Illumina base caller have been shown to be of limited use for the identification of actual sequence errors for 16S rRNA gene amplicon studies ^17, 38^. Additionally, these quality scores only check for errors that occurred during sequencing, but do not account for other sources of error, such as PCR amplification, whereas quality filtering by abundance is sensitive to any source of error. Moreover, the application of global parameters ( *e.g.* average Phred score) ignores that error is sequence-specific, and hence some sequences could be affected more than others. If a species specific amplicon is more prone to PCR or sequencing errors, the relative abundance of that particular species will be underestimated. To compensate for this potential bias, discarded reads are clustered to the OTUs with one mismatch.

Finally, all OTUs are subjected to non-reference based chimera checking according to the following principle: given three OTUs named A, B and C, C will be considered a chimera when the following conditions are satisfied: C and A 5’ reads are identical, C and B 3’ reads are identical and both OTUs, A and B, are at least twice as abundant as OTU C. A complete overview of the number of sequences retained in both pipelines, *i.e.* NG-Tax and QIIME, as well as the final number of OTUs, is provided in Dataset 1.

***Taxonomic assignment.*** In the current version of NG-Tax, taxonomy is assigned to OTUs utilizing the USEARCH algorithm ^22^ and the Silva 128 SSU Ref database, containing 1.922.223 unique full length 16S rRNA gene sequences. To ensure maximum resolution and avoid the risk of errors due to clustering-associated flaws ( *e.g.* reference sequence error hotspots, overrepresentation of certain species and lack of robustness in cluster formation by clustering algorithms), we use a non-clustered database. To speed up the procedure by several orders of magnitude, 16S rRNA gene sequences from the reference database are trimmed to the amplified region using the primers as a guide. For each OTU, a taxonomic assignment is retrieved at six different identity thresholds levels (100%, 98%, 97%, 95%, 92% and 90%) and at two taxonomic levels (genus and family). The final taxonomic label is determined by the assignments that show concordance at the highest taxonomic resolution. Similar dynamic thresholds are used in rtax ^39^.

## Validation

### Datasets

Our main objective was to develop a pipeline that accurately reproduces the composition of the synthetic MCs and also reduces the impact of experimental choices. To achieve this goal, four synthetic communities of varying complexity were created, consisting of full length16S rRNA gene amplicons of phylotypes (PTs) associated with the human GI-tract ( Dataset 1). This specific setup limited the likelihood of overfitting to a particular OTU composition or distribution and allowed us to assess (1) the quantification potential, (2) noise floor and (3) the effect of richness and diversity on quality filtering parameters, thus ensuring a higher fidelity with biological samples than by using a single MC. As a reference, to assess the quality of the taxonomic classifications, full length sequences for all PTs were obtained through Sanger sequencing. Expected MCs were created in silico by trimming the full length sequences to the sequenced region. MC1 and MC2 consisted of equimolar amounts of 17 and 55 PTs, respectively. MC3 contained 55 PTs in staggered concentrations typical for the human GI-tract, and MC4 included 50 PTs with relative abundances ranging between 0.001 and 2.49%. To account for pipetting errors, each of the four MCs was produced in triplicate. These 12 MC templates were used to sequence the MCs with different conditions that cover most of the technical bias associated with 16S rRNA gene amplicon studies reported in literature. To this end, we 1) targeted either region V4 or region V5-V6, 2) used four PCR protocols differing in the number of PCR cycles and reaction volumes 3) PCR products were analysed in three different sequencing runs and in seven different libraries, and 4) two different library preparation protocols (with and without an extra amplification of 10 cycles) were applied ( Dataset 1). In addition the sequencing depth ranged from 1911 to 334613 reads per sample ( Dataset 1).

### NG-Tax classification of short reads versus full length classification

**Figure 2.**
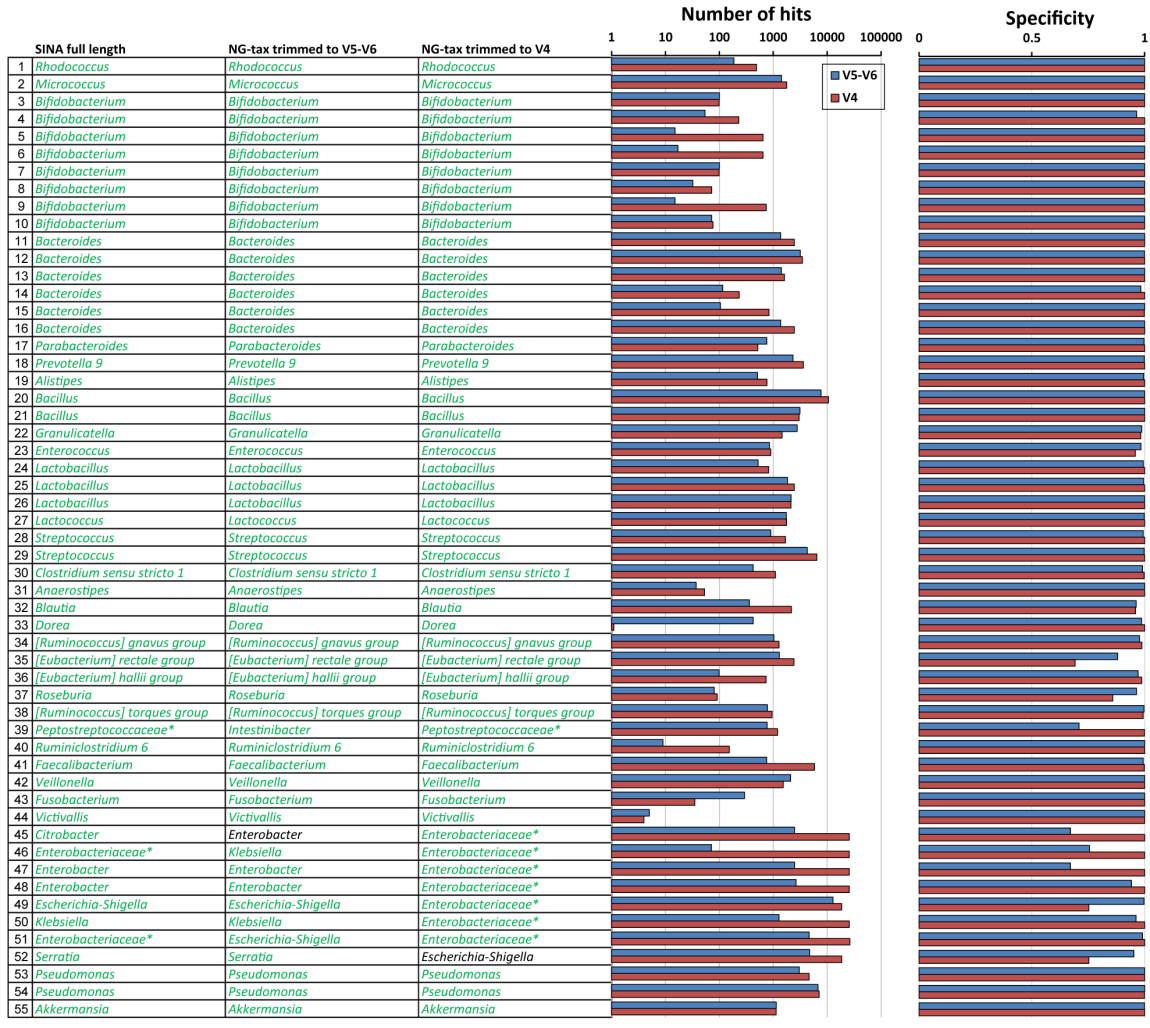
NG-Tax Assignment quality of the 55 MC phylotypes. Three taxonomic assignments are shown: RDP full length, NG-Tax V5-V6 trimmed and NG-Tax V4 trimmed. If NG-Tax assignments are in agreement with SINA full length assignment, that classification is shown in green. Assignment specificity (the fraction of hits with an identical label) and the total number of hits supporting this taxonomic label are shown in blue for V5-V6 region and in red for V4 region

To evaluate the accuracy and reproducibility of taxonomic classification using a low information content of ~140 nt compared to a maximum information content of ~1500 nt, we compared the NG-Tax classification of all 55 reference sequences trimmed to V4 and V5-V6, with a classification of the corresponding full length reference sequences using the Silva Incremental Aligner (SINA) with SILVA taxonomy ^40^ ( Figure 2). At family level, all three classifications (i.e. full length, V4 and V5-V6) were in complete concordance for all phylotypes. Correspondingly, the consistency at genus level was very high. Only five phylotypes for V4 that belong to the poorly classified family Enterobacteriaceae, attained higher resolution using the full length sequences. In turn, for *Intestinibacter* (PT39, V5-V6) and *Klebsiella* (PT46, V5-V6), a higher resolution was attained with short reads due to the high specificity of the hypervariable region, which can be overshadowed when using the full length sequence. Lastly, only two assignment at genus level, both Enterobacteriaceae (PT52, V4 and PT45, V5-V6) were incongruent between classification of the short and full length sequences. Overall, the V5-V6 amplicons outperformed the V4 amplicons because this region allowed for differentiation between Enterobacteriaceae and even attained a higher resolution than full length sequences for some sequences. The average taxonomic specificity (percentage of hits with an identical taxonomic label) for all reference phylotypes was 97.78% for both regions with an average of 4837 and 1688 hits for regions V4 and V5-V6, respectively. The high specificity and high number of hits at very high identity thresholds, combined with the fact that the vast majority of V4 and V5-V6 based assignments matched to each other as well as to the full ength classification, testifies for the reliability and quality of the assignments.

### Observed versus expected microbial profiles

**Figure 3.**
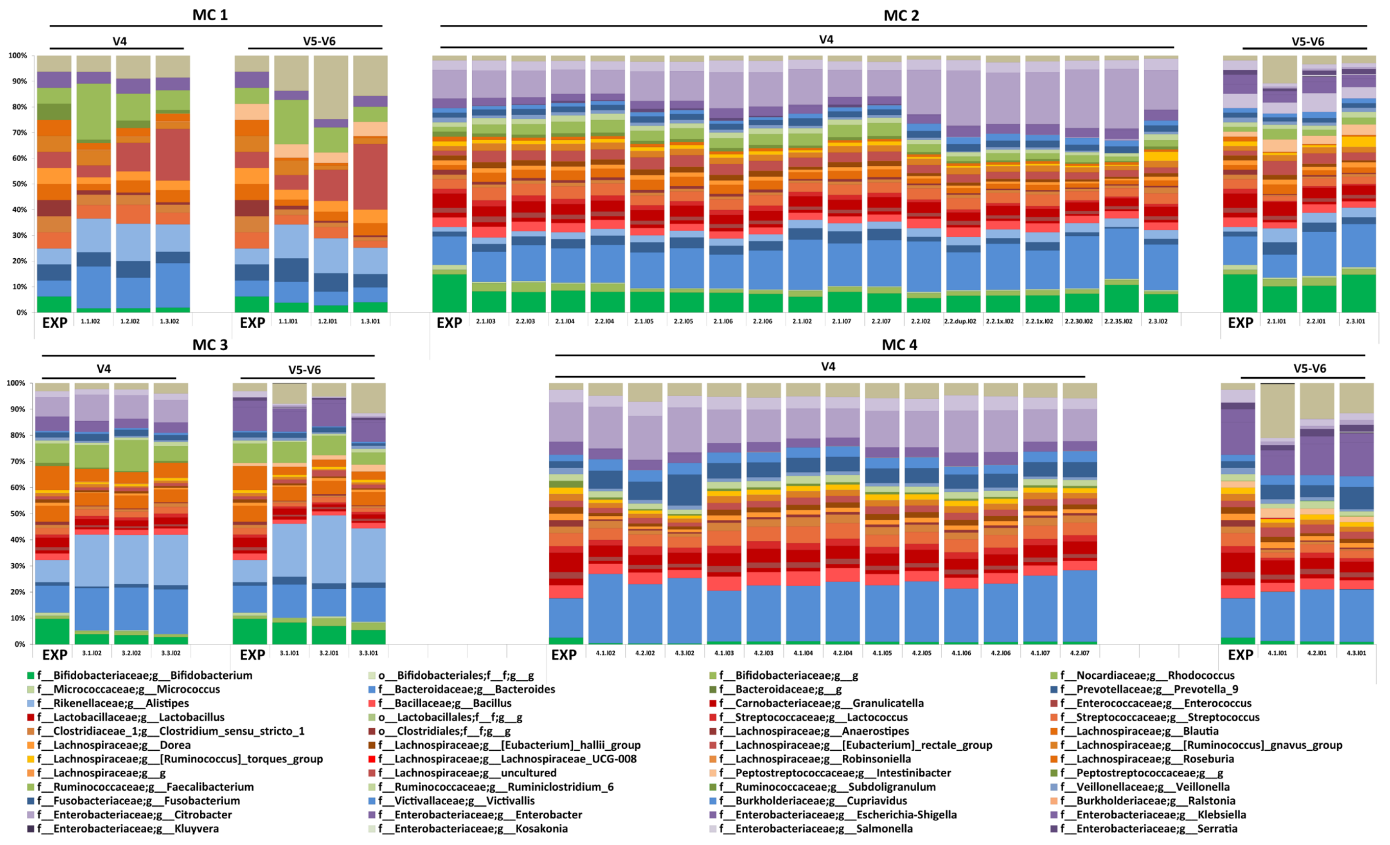
Observed composition of all MCs compared with the expected ones (EXP) for both regions obtained with NG-Tax.

**Figure 4.**
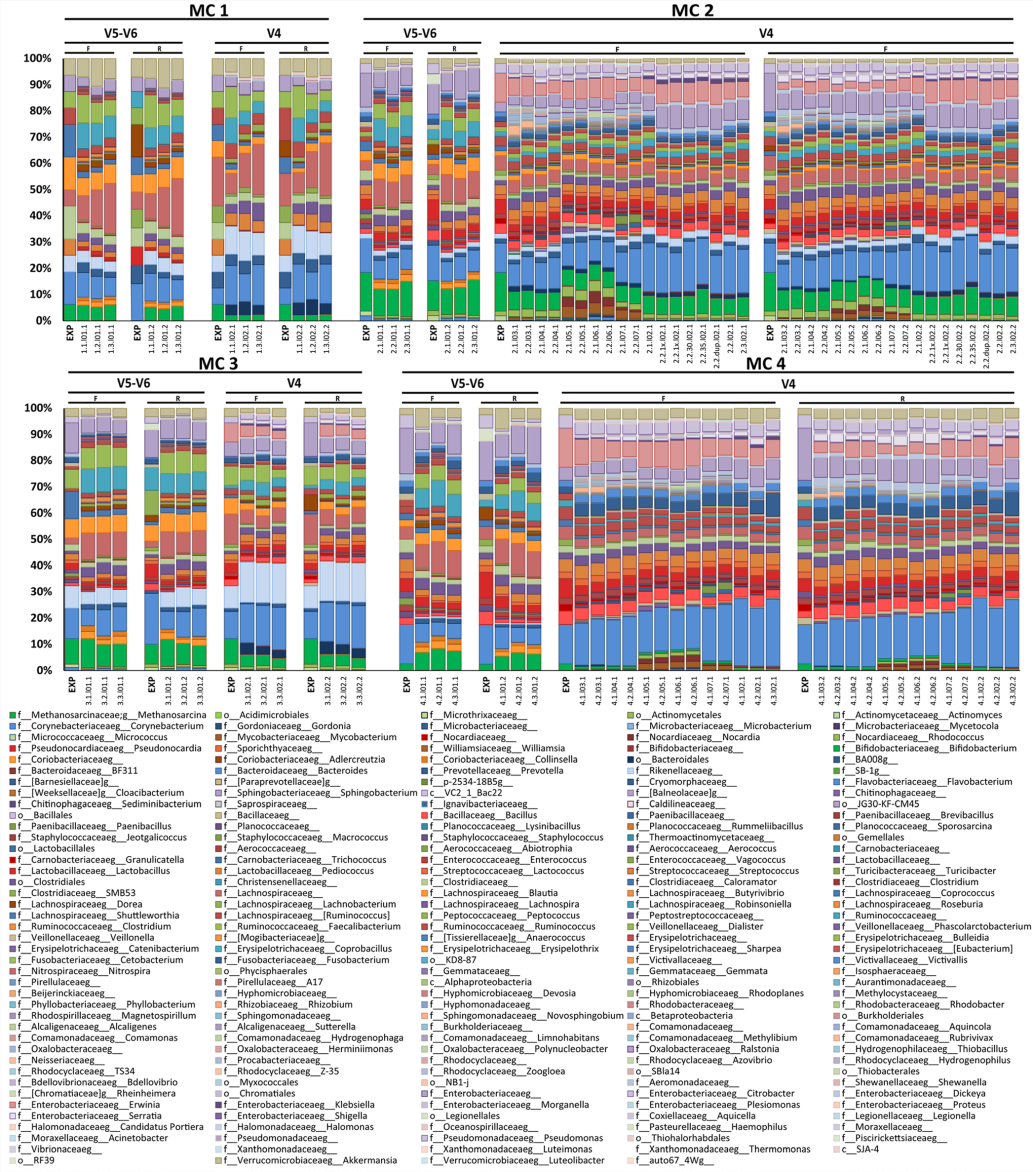
Observed composition of all MCs compared with the expected ones (EXP) for both regions and each read separately obtained with QIIME.

To assess the ability to reproduce the expected composition of the MCs we benchmarked NG-Tax with QIIME, a common 16S rRNA gene amplicon analysis pipeline. Table 1 shows the comparison between NG-Tax and QIIME per region and taxonomic rank with the percentage of classified reads, the amount of spurious taxa and the total percentage of misclassified reads. The number of classified sequences without considering their accuracy is higher for NG-Tax at each taxonomic rank, with relatively small differences with QIIME. However, the number and percentage of spurious reads is considerably higher for QIIME with some regions generating an average of 18.65% incorrectly assigned reads at the genus level, compared to 0.3% for NG-Tax. Consequently, NG-Tax ensured excellent reproduction of the expected profiles ( Figure 3), while the QIIME profiles suffered from high a high fraction of poorly classified and spurious OTUs ( Table 1, Figure 4).

**Table 1.** Performance of NG-Tax and QIIME at different taxonomic levels for region V4 and V5-V6. Classified reads are defined as reads mapped to a sequence for which a genus, family or order level classification is given, without considering accuracy. The percentage represents the average over all samples. Spurious taxa are taxonomic classes not included in the MCs. The percentage of spurious reads is the percentage of total reads in the misclassified classes. F: forward read, R: reverse read.

**V4**								
	Classified reads (%)		Spurious taxa (#)			Spurious reads (%)		
	NG-Tax	QIIME	NG-Tax	QIIME F & R		NG-Tax	QIIME F & R	
**Genus**	86.23	60.66	4	110	110	0.19	9.02	15.05
**Family**	99.97	96.23	1	82	81	0.19	8.43	6.42
**Order**	100	100.00	1	49	47	0.19	6.40	5.47
**V5-V6**								
	Classified reads (%)		Spurious taxa (#)			Spurious reads (%)		
	NG-Tax	QIIME	NG-Tax	QIIME F & R		NG-Tax	QIIME F & R	
**Genus**	99.23	69.99	5	53	51	0.28	13.42	18.65
**Family**	99.89	93.63	0	29	29	0.00	9.64	12.05
**Order**	100	99.81	0	15	17	0.00	6.33	6.45

### Observed versus expected diversity

**Figure 5.**
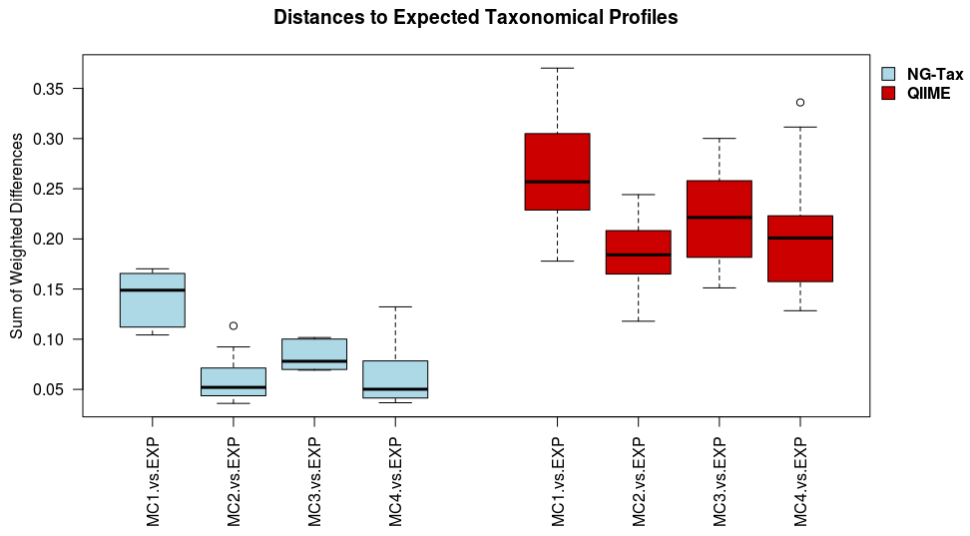
Distances to expected taxonomical profiles. NG-Tax results are depicted in blue and QIIME in red.

To quantify the distances to the expected profiles, the sum of weighted differences were calculated. Given two taxonomical profiles x and y, for each taxon i, we defined the difference in abundance as difi(x,y)=(xi –yi) and a weighting factor wi as wi(x,y)=(xi –yi)/avg(xi + yi). The weighted difference was obtained by multiplying the difference in abundance by its weighing factor. This weighing factor is used to take the relative change as well the absolute change into account, because a 1% absolute change becomes a 200% or 20% relative change depending on whether the expected abundance is 0.5% or 5%, respectively. Distances to the expected profile were significantly lower for NG-Tax (p<1e-4) compared to QIIME using a two-tailed t-test ( Figure 5 and Dataset 1).

One template, PT17 ( *Parabacteroides*), triggered so much sequencing error in the V4 region that it was rendered undetectable although it was amplified by the primers ( Supplementary Figure 1). Therefore, to test both pipelines without this sequencing anomaly, it was removed from the analysis.

Richness and diversity measures are important for understanding community complexity and dynamics. Among these measures, α-diversity is defined as the diversity within a sample, which is often estimated based on the abundance distribution (evenness) and number (richness) of species, whereas β-diversity is defined as the partitioning of diversity among communities. The ability of researchers to quantify richness and diversity hinges on an accurate assessment of the composition of these communities ^41^. For microbial communities, this has been particularly challenging, as none of the existing molecular microbial ecology methods normally captures more than a small proportion of the estimated total richness in most microbial communities ^42^. For deep sequencing based approaches, filtering strategies that remove low-abundance reads make it impossible to apply richness estimation metrics such as the Chao1 index and the ACE coverage estimator, because low-abundance read counts are included in their calculations. Conversely, richness estimates based on unfiltered datasets are unlikely to be accurate, if many of the reads actually represent PCR and/or sequencing errors ^16^. In contrast to purely OTU-based methods, divergence-based methods account for the fact that not all species within a sample are equally related to each other, considering two communities to be similar if they harbour the same phylogenetic lineages, even if the species representing those lineages in each of the communities are different. Consequently, these methods are more powerful than purely OTU-based methods, because similarity in 16S rRNA gene sequence often correlates with phenotypic similarity in key features such as metabolic capabilities. An added benefit is that small errors that are likely due to unfiltered sequencing errors, are punished less severely because OTUs that are only a few nt distant from each other due to error are still closely related using divergence based indices ^43^. Therefore, these indices probably provide a better estimate of the true diversity for data generated by high throughput next generation technology sequencers.

**Figure 6.**
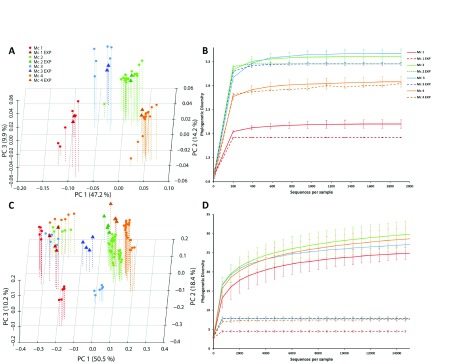
PCoA using Weighted Unifrac of all sequenced and expected MCs as obtained after processing of data using NG-Tax ( **A**) and QIIME ( **C**). Darker colored triangles represent the expected composition while lighter colored circles represent sequenced samples. **B/D.** Rarefaction curves of PD for all MCs and their expected counterparts for NG-Tax ( **B**) and QIIME ( **D**). Dashed lines represent the expected composition while solid lines represent sequenced samples.

Because the aim of NG-Tax is to enhance the biological signal as much as possible by minimizing the impact of any technical aspect, divergence-based α-diversity (Phylogenetic Diversity (PD) ^44^) and β-diversity (Unifrac ^41^) metrics were used to visualize the diversity within and between MCs ( Figure 6). The results obtained with QIIME suffered from all of the previously described technological artifacts. The MCs clustered by primer pair instead of MC, and within each cluster the structure, *i.e*. the position of MCs relative to each other, was different. More importantly, the true biological variation depicted by the expected composition was reproduced by neither primer pair ( Figure 6C). Based on these results not only the Principle Coordinates Analysis (PCoA) based conclusions would have been different for both primer pairs, but also the differences in taxonomic classification could lead to significant changes in identified biomarkers, in line with what has previously been observed by He and co-workers ^30^ as well as Edgar ^43^. Here we show that replicability within a variable region was attained. The more important reproducibility, however, *i.e.* the corroboration of findings by reproduction in different independent setups that use *e.g*. different primers, was not. This is an important observation because biological findings should be insensitive to independent methodologies ^11^. In line with the above, also the observed α-diversity (PD) was found highly inflated and the biological order was not reproduced ( Figure 6D). In contrast, NG-Tax provided a clear separation of samples by MC type and their representative expected samples regardless of variable region, PCR protocol, sequencing run, library and sequencing depth. These results are remarkable, given the biases associated with each of these categories and the difference in resolution between the two regions ( Figure 6A). Moreover, MC2, MC3 and MC4 were very similar, sharing the same genera and largely the same phylotypes, only differing in relative distribution ( Dataset 1). Correspondingly, rarefaction curves for α-diversity ( Figure 6B) showed excellent reproduction of the true diversity. A perfect overlap cannot be achieved since the expected MCs do not account for sequencing or PCR errors, and these factors cannot be completely removed from real sequencing data. Results for α-diversity and β-diversity using different metrics can be found in Dataset 1.

**Figure 7.**
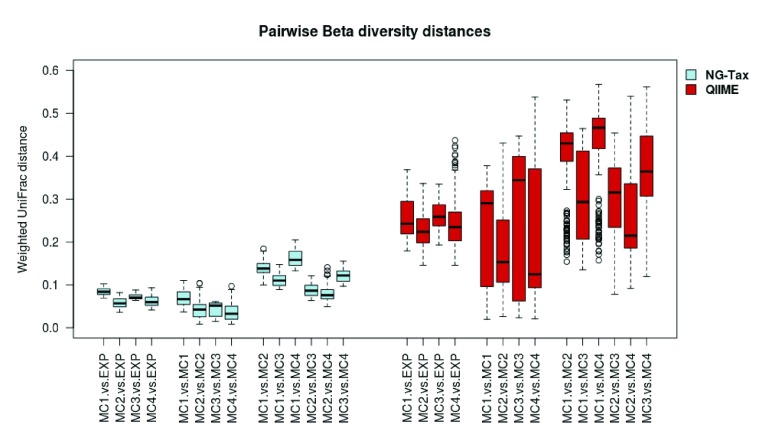
Pairwise Weighted UniFrac distances. NG-Tax results are depicted in blue and QIIME in red.

Small distances to expected MCs show the accuracy of NG-Tax, reproducibility on the other hand can be evaluated by the within MCs distances and also by the dispersion of the between MCs distances ( Figure 7). Distances to the expected MCs, within MC distances and dispersion of the between MCs distances were significantly (p<1e-10) lower for NG-Tax ( Dataset 1). K-means cluster prediction using within groups sum of squares, predicted 2 groups for QIIME ( Supplementary Figure 2) and the correct 4 groups for NG-Tax ( Supplementary Figure 3) ^45^.

Raw data of NG-Tax pipeline for analysis of 16S rRNA amplicons from complex biome Click here for additional data file. Copyright: © 2018 Ramiro-Garcia J et al. 2018 Data associated with the article are available under the terms of the Creative Commons Zero "No rights reserved" data waiver (CC0 1.0 Public domain dedication).

## Conclusions

An increasing number of studies have shown that the chosen methodology rather than the natural variance is responsible for the greatest variance in microbiome studies ^6– 12^. Some authors raised their concern when comparing data generated using different strategies ^5^, which basically suggests that true reproducibility ( *i.e.* using different approaches and drawing the same biological conclusions) is unattainble. This is an alarming observation since studies are often used to identify biomarker organisms, associated with certain host phenotypes (often comparing a diseased state to a healthy state), yet the use of different primers might show different biomarkers ^6, 8, 17, 29, 30, 35^. So far, neither currently available pipelines nor taxonomic classifiers have been able to efficiently reduce the noise in this type of data. Nevertheless, in properly de-noised datasets, taxonomical profiles, richness and diversity should be close to the expected values and the abundance of unassigned and poorly assigned reads should be low except when dealing with largely unexplored environments that are not sufficiently covered yet by the reference databases. At lower noise levels different variable regions should yield similar conclusions with small variations due to region specific resolution, and minor changes in the experiment should still deliver the same biological conclusions. Here we presented NG-Tax, an improved pipeline for 16S rRNA gene amplicon sequencing data, which continues to be a backbone in the analysis of microbial ecosystems. Several novel steps ensure much needed improved robustness against errors associated with technical aspects of these studies, such as PCR protocols, choice of 16S rRNA gene variable region and variable rates of sequencing error ^5, 6, 12^. The commonly reported problems such as many un- or poorly classified OTUs, inflated richness and diversity, taxonomic profiles that do not match the expected ones, region dependent taxonomic classification and results being highly dependent on minor changes in the experimental setup have been tackled with NG-Tax. Despite the short read length (~140 nt) and all technical biases, the average taxonomic assignment specificity for the phylotypes included in the MCs was 97.78%. In addition, 89,43.% of the reads could be assigned to at genus level and 99.95% to at least family. Spurious genera represented only 0.21% of the reads per sample. More importantly, rarefaction curves and PCoA plots confirmed improved performance of NG-Tax with respect to clustering based on biology rather than technical aspects, such as sequencing run, library or choice of 16S rRNA gene region. Therefore, NG-Tax represents a method for 16S rRNA gene amplicon analysis with improved qualitative and quantitative representation of the true sample composition. Additionally, the high robustness against technical bias associated with 16S rRNA gene amplicon studies will improve comparability between studies and facilitate efforts towards standardization.

## Methods

### Primers

Primer pairs 515F (5’-GTGCCAGCMGCCGCGGTAA) - 806R (5’-GGACTACHVGGGTWTCTAAT) and BSF784F (5’-RGGATTAGATACCC) - 1064R (5’-CGACRRCCATGCANCACCT) have been previously reported for amplification of the V4 ^17^ and V5- V6 ^6^ regions of the bacterial 16S rRNA gene, respectively. They were selected based on 1) experimental validation, 2) taxonomic coverage of the relevant ecosystem ( Supplementary Figure 4) and 4) adherence to specific rules associated with the sequencing platform, such as a maximum amplicon size of <500 nt. Unless noted otherwise all primers were ordered at Biolegio (Nijmegen, Netherlands).

### Barcoding strategy

At the time of sequencing Illumina’s Hiseq2000 allowed for multiplexing of up to 12 samples per lane using an index or barcode read provided by Illumina. To achieve optimal sample throughput and phylogenetic depth, 70 primers containing a custom designed 8nt barcode were developed to combine with the Illumina barcodes to reach a maximum throughput of 12×70 samples per lane. Each set of 70 barcoded samples are referred to as “library”. Low diversity samples, such as 16S rRNA gene amplicons, can lead to problems with base calling due to overexposure of fluorescent labels. Therefore, the set of 70 barcodes was specifically designed to possess an equal base distribution over their complete length. Additionally, to avoid differential amplification, a two-base “linker” sequence that is not complementary to any 16S rRNA sequence at the corresponding position, from a database that contains 1132 phylotypes associated with the Human GI tract ^46^, was inserted between the primer and barcode. The resulting set of 70 barcoded primers was checked for avoidance of secondary structure formation within or between primers ( *i.e*., primer-dimers) or between barcodes and primers, using PrimerProspector ^47^.

### Mock communities

All MCs were mixed in triplicate to account for pipetting error. These MCs ranged from 17–55 species in both equimolar and staggered compositions. One MC contained members at very low abundances of 0.1, 0.01 and 0.001% ( Dataset 1). Amplicons were generated either from cloned 16S rRNA gene amplicons, isolates available in the local culture collection of the Laboratory of Microbiology, Wageningen University, or strains ordered from DSMZ and cultured according to DSMZ recommendations, after which genomic DNA was isolated using the Genejet genomic DNA isolation kit (Thermo fisher scientific AG, Reinach, Zwitserland). A 16S rRNA gene specific PCR was performed using the universal primers 27F (5’-GTTTGATCCTGGCTCAG) - 1492R (5’-GGTTACCTTGTTACGACTT) to obtain full length amplicons of which size and concentration were checked on a 1% agarose gel and which were column purified and quantified with the Qubit 2.0 fluorometer, and dsDNA BR assay kit (Invitrogen, Eugene, USA). Amplicons were mixed in the MCs to obtain the specified relative abundances. High quality full length reference sequences of all MC members were obtained by Sanger sequencing at GATC Biotech AG (Constance, Germany) with sequencing primers 27F - 1492R in order to confirm their identity. The MCs were diluted 10 ^3^-fold and subsequently used as templates in PCRs for the generation of barcoded PCR products.

## Barcoded PCR

Unless noted otherwise, each sample was amplified in triplicate using Phusion hot start II high fidelity polymerase (Thermo fisher scientific AG), checked for correct size and concentration on a 1% agarose gel and subsequently combined and column-purified with the High pure PCR cleanup micro kit (Roche diagnostics, Mannheim, Germany). Forty μl PCR reactions contained 28.4 μL nucleotide free water (Promega, Madison, USA), 0.4 μL of 2 U/μl polymerase, 8 μL of 5× HF buffer, 0.8 μl of 10 μM stock solutions of each of the forward (515F) and reverse (806R) primers, 0.8 μL 10mM dNTPs (Promega) and 0.8 μL template DNA (10 ^3^ × diluted 200 ng/μl stock). Reactions were held at 98°C for 30 s and amplification proceeding for 25 cycles at 98°C for 10 s, 50°C for 10 s, 72°C for 10 s and a final extension of 7 min at 72°C. Purified amplicons were quantified using Qubit. For primer pair BSF784F-1064R the thermal cycling conditions were identical to those detailed above except that the annealing temperature was 42°C. To quantify noise generated by the PCR protocol, several reactions were performed with 30 or 35 cycles and 1× 100μl reaction instead of pooling 40μl in triplicate ( Dataset 1).

A composite sample for sequencing was created by combining equimolar amounts of amplicons from the individual samples, followed by gel purification and ethanol precipitation to remove any remaining contaminants. The resulting libraries were sent to GATC Biotech AG for sequencing on an Illumina Hiseq2000 instrument.

## Sequence analysis with QIIME

We have used QIIME to benchmark NG-Tax. Illumina fastq files were de-multiplexed, quality filtered and analyzed using QIIME (v. 1.9) ^13^ with closed reference OTU picking, using default settings and quality parameters as previously reported ^12^.

## NG-tax pipeline and user manual

The NG-tax pipeline, user manual and files and code to reproduce the presented results, are available for download at http://github.com/JavierRamiroGarcia/NG-Tax.

## Abbreviations

**rRNA:** ribosomal RNA; **MC:** Mock Community; **OTU:** Operational Taxonomic Unit; **PT:** Phylotype; **RDP:** Ribosomal Database Project; **RDPc:** RDP classifier; **PD:** Phylogenetic Diversity; **PCoA:** Principle Coordinates Analysis

## Data availability

The data referenced by this article are under copyright with the following copyright statement: Copyright: © 2018 Ramiro-Garcia J et al.

Data associated with the article are available under the terms of the Creative Commons Zero "No rights reserved" data waiver (CC0 1.0 Public domain dedication). 

F1000Research: Dataset 1. Raw data of NG-Tax pipeline for analysis of 16S rRNA amplicons from complex biome, https://doi.org/10.5256/f1000research.9227.d226015 ^48^

Sequence data have been deposited in the European Nucleotide Archive ^49^, accession number [ENA:PRJEB11702]) http://www.ebi.ac.uk/ena/data/view/PRJEB11702 (amplicon sequencing data for all 49 samples) and [ENA:LN907729-LN907783]) (full length 16S rRNA gene sequences for all 55 PTs).

## Supplementary material

**Supplementary Figure 1. A) Nucleotide distribution of PT17 (Parabacteroides) for each of the four primers. Positions under the black segment are fixed and specific for PT17 preventing the inclusion of sequences belonging to a different PT. B) Percentage of 10 most abundant sequences for PT17 obtained with each of the primers.**

PT17 (Parabacteroides) presented a sequencing anomaly in the reverse V4 region (primer R806) ( Supplementary Figure 1A). From positions 50 to 67 this region had higher error rate than the other three regions. The noise generated from this anomaly masked the biological signal rendering PT17 undetectable. In fact the most abundant sequence represented less than 0.45% of the total reads, while for the other three regions the most abundant sequence represented more than 80% ( Supplementary Figure 1B). We decided to remove the sequences belonging to PT17 from V5-V6 samples to avoid region clustering due to the presence of PT17. Our intention in this study was to test region performance under conditions in which sequencing anomalies like the one showed in Supplementary Figure 1 are not present.

**Supplementary Figure 2. K-means cluster prediction for QIIME results.**

The number of clusters is chosen using the “elbow criterion”. When the marginal gain of variance explained drops the line bents indicating the number of clusters.

**Supplementary Figure 3. K-means cluster prediction for NG-Tax results.**

The number of clusters is chosen using the “elbow criterion”. When the marginal gain of variance explained drops the line bents indicating the number of clusters.

**Supplementary Figure 4. Taxonomic coverage of primers.**

Forward (left bars) and reverse (right bars) primer coverage of the major bacterial phyla associated with the human GI tract using RDP’s probematch program with one mismatch allowed.

**Supplementary Figure 5. Beta-diversity measures for NG-Tax results.**
