# Supplementary figures and images for: NG-Tax, a highly accurate and validated pipeline for analysis of 16S rRNA amplicons from complex biomes
Source: F1000Res. 2018 Nov 23;5:1791. Originally published 2016 Jul 22. [Version 2] doi: 10.12688/f1000research.9227.2 (PMC6419982; doi:10.12688/f1000research.9227.2)

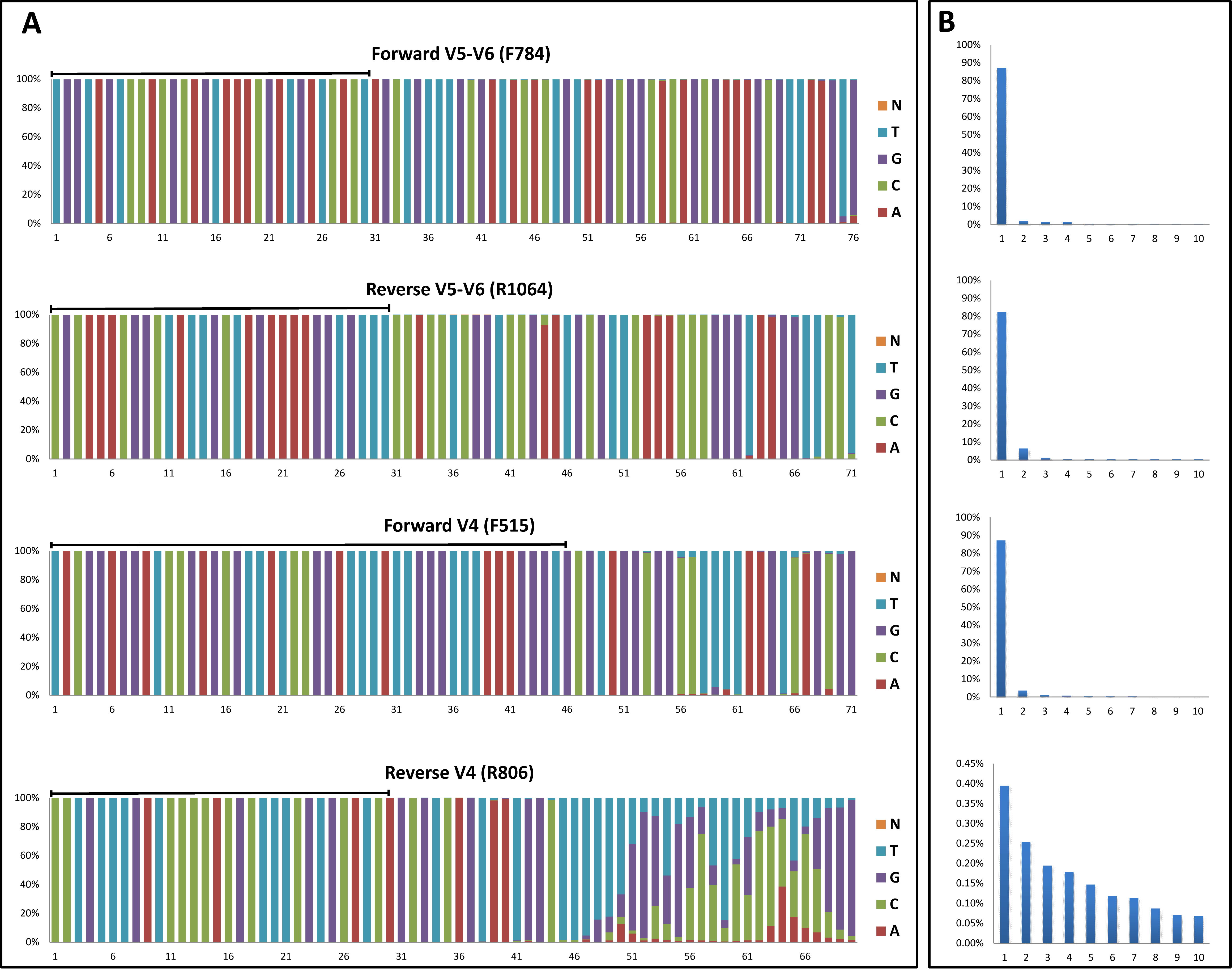

Supplement: Supplementary file 2 [file f1000research-5-18667-s0001.tgz › ea7bd2dd-3067-4059-983e-986fafe7d148_Supplementary_Figure_1._Parabacteroides_motif.tif]

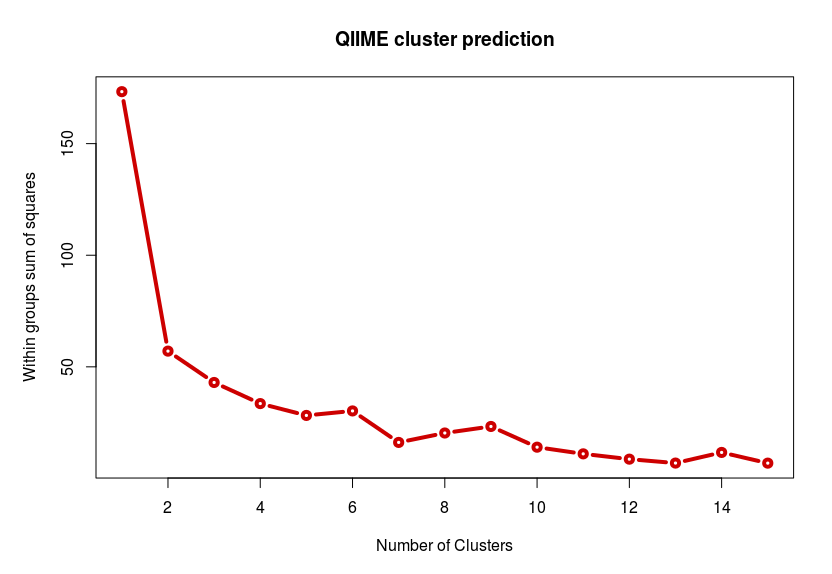

Supplement: Supplementary file 3 [file f1000research-5-18667-s0002.tgz › 58b7a2d7-582d-4aa9-8d39-476eab88ab3b_Supplementary_Figure_2._K-means_cluster_prediction_QIIME.tiff]

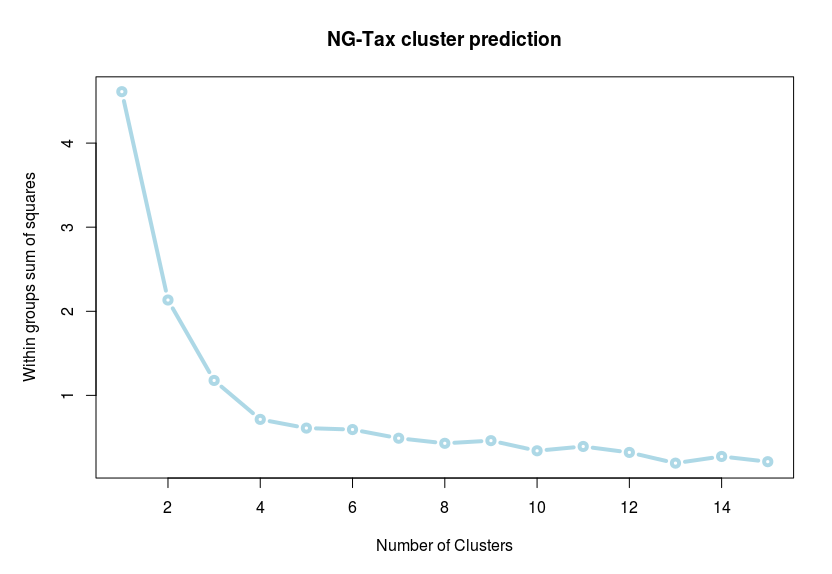

Supplement: Supplementary file 4 [file f1000research-5-18667-s0003.tgz › 826f6688-22b0-4b23-9db5-01bd29ff83c1_Supplementary_Figure_3.__K-means_cluster_prediction_NG-Tax.tiff]

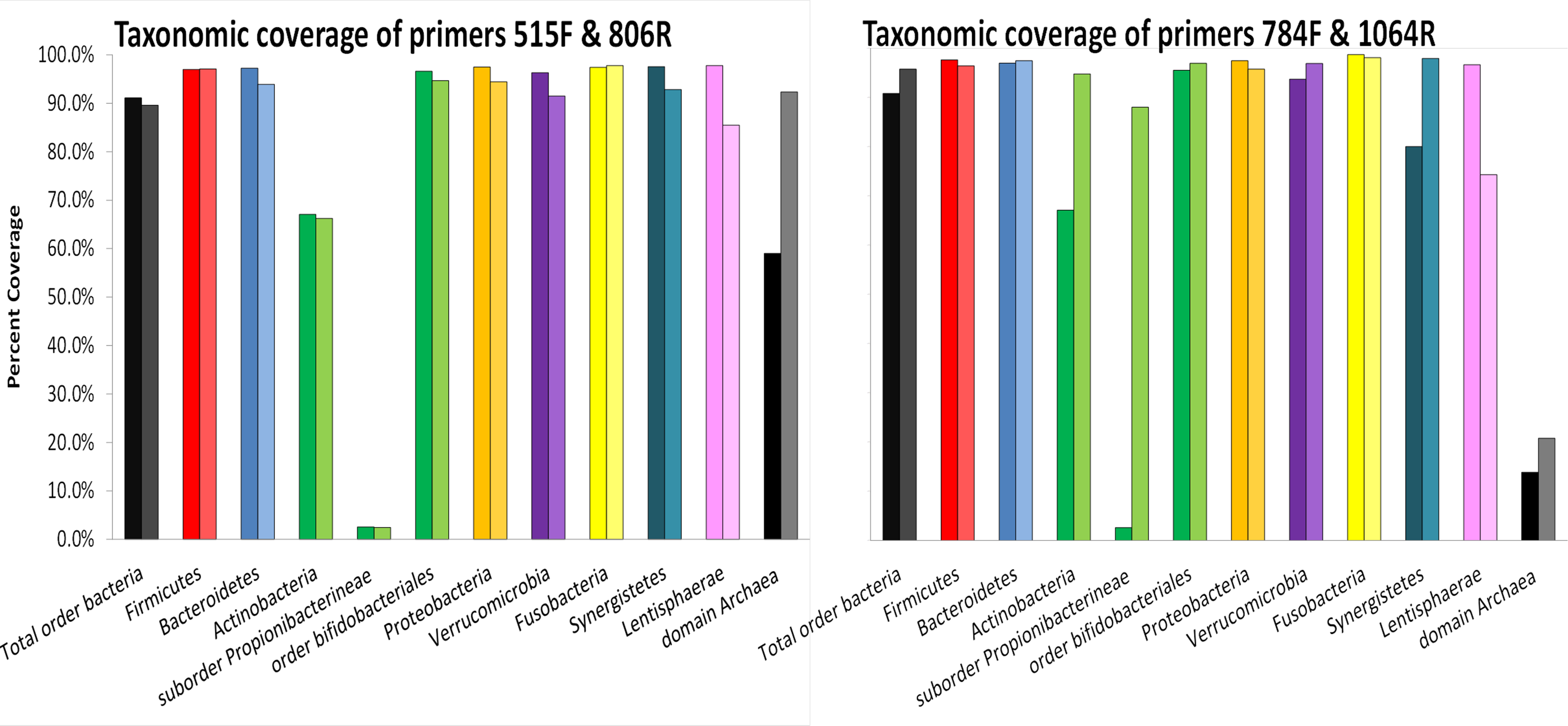

Supplement: Supplementary file 5 [file f1000research-5-18667-s0004.tgz › 6dd926f3-0e17-420b-bc69-af7dcbd654c9_Supplementary_Figure_4._Primer_coverage.tif]

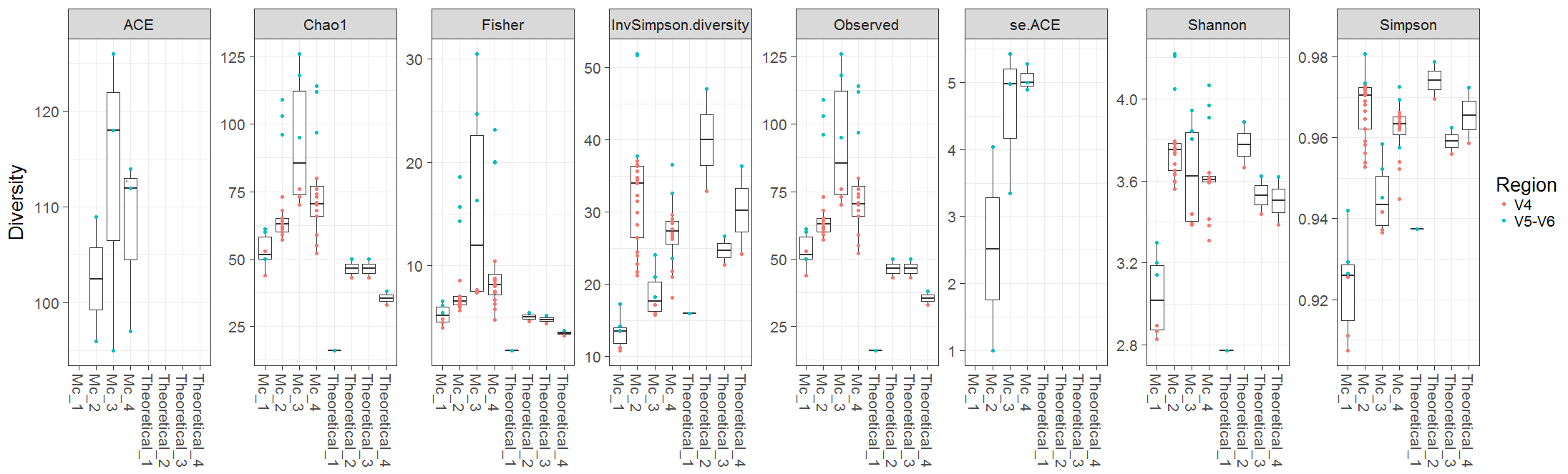

Supplement: Supplementary file 6 [file f1000research-5-18667-s0005.tgz › 37ccc361-29c7-4159-94f2-8c5c888f1ea0_Supplementary_Figure_5._Alpha_diversity_other_metrics.tiff]
