# Supplementary material for: NG-Tax, a highly accurate and validated pipeline for analysis of 16S rRNA amplicons from complex biomes
Source: F1000Res. 2018 Nov 23;5:1791. Originally published 2016 Jul 22. [Version 2] doi: 10.12688/f1000research.9227.2 (PMC6419982; doi:10.12688/f1000research.9227.2)
Supplement: Raw data of NG-Tax pipeline for analysis of 16S rRNA amplicons from complex biome [file f1000research-5-18667-s0000.tgz › 29a888b8-2acd-4783-bd99-cd03cb48685b_Dataset_1_v2.zip!/Dataset 1/Dataset 1.10 Significance tests NG-Tax versus QIIME.docx]

```
1. t-tests means NG-Tax taxa distances versus QIIME taxa distances


> t.test(taxa_d_ngtax$MC1.vs.EXP,taxa_d_qiime_default$MC1.vs.EXP,conf.level=0.99)

	Welch Two Sample t-test
```

```
data:  taxa_d_ngtax$MC1.vs.EXP and taxa_d_qiime_default$MC1.vs.EXP
t = -6.18, df = 16, p-value = 1.321e-05
alternative hypothesis: true difference in means is not equal to 0
99 percent confidence interval:
 -0.18220417 -0.06525177
sample estimates:
mean of x mean of y
0.1415997 0.2653277

> t.test(taxa_d_ngtax$MC2.vs.EXP,taxa_d_qiime_default$MC2.vs.EXP,conf.level=0.99)

	Welch Two Sample t-test

data:  taxa_d_ngtax$MC2.vs.EXP and taxa_d_qiime_default$MC2.vs.EXP
t = -17.936, df = 59.574, p-value < 2.2e-16
alternative hypothesis: true difference in means is not equal to 0
99 percent confidence interval:
 -0.1439976 -0.1067919
sample estimates:
 mean of x  mean of y
0.05810897 0.18350372

> t.test(taxa_d_ngtax$MC3.vs.EXP,taxa_d_qiime_default$MC3.vs.EXP,conf.level=0.99)

	Welch Two Sample t-test

data:  taxa_d_ngtax$MC3.vs.EXP and taxa_d_qiime_default$MC3.vs.EXP
t = -9.1152, df = 14.36, p-value = 2.371e-07
alternative hypothesis: true difference in means is not equal to 0
99 percent confidence interval:
 -0.18367380 -0.09350074
sample estimates:
 mean of x  mean of y
0.08277303 0.22136030

> t.test(taxa_d_ngtax$MC4.vs.EXP,taxa_d_qiime_default$MC4.vs.EXP,conf.level=0.99)

	Welch Two Sample t-test

data:  taxa_d_ngtax$MC4.vs.EXP and taxa_d_qiime_default$MC4.vs.EXP
t = -11.818, df = 45.967, p-value = 1.562e-15
alternative hypothesis: true difference in means is not equal to 0
99 percent confidence interval:
 -0.1738622 -0.1094477
sample estimates:
 mean of x  mean of y
0.06274908 0.20440401


2. t test means beta diversity distances to expected. NG-Tax versus QIIME default parameters.


> t.test(bdiv_ngtax$MC1.vs.EXP,bdiv_qiime_default$MC1.vs.EXP,conf.level=0.99)

	Welch Two Sample t-test

data:  bdiv_ngtax$MC1.vs.EXP and bdiv_qiime_default$MC1.vs.EXP
t = -23.009, df = 57.21, p-value < 2.2e-16
alternative hypothesis: true difference in means is not equal to 0
99 percent confidence interval:
 -0.1921066 -0.1522303
sample estimates:
 mean of x  mean of y
0.08415392 0.25632239

> t.test(bdiv_ngtax$MC2.vs.EXP,bdiv_qiime_default$MC2.vs.EXP,conf.level=0.99)

	Welch Two Sample t-test

data:  bdiv_ngtax$MC2.vs.EXP and bdiv_qiime_default$MC2.vs.EXP
t = -44.864, df = 207.14, p-value < 2.2e-16
alternative hypothesis: true difference in means is not equal to 0
99 percent confidence interval:
 -0.1773639 -0.1579340
sample estimates:
 mean of x  mean of y
0.05850063 0.22614958

> t.test(bdiv_ngtax$MC3.vs.EXP,bdiv_qiime_default$MC3.vs.EXP,conf.level=0.99)

	Welch Two Sample t-test

data:  bdiv_ngtax$MC3.vs.EXP and bdiv_qiime_default$MC3.vs.EXP
t = -39.953, df = 57.977, p-value < 2.2e-16
alternative hypothesis: true difference in means is not equal to 0
99 percent confidence interval:
 -0.2026403 -0.1773122
sample estimates:
 mean of x  mean of y
0.07230663 0.26228289

> t.test(bdiv_ngtax$MC4.vs.EXP,bdiv_qiime_default$MC4.vs.EXP,conf.level=0.99)

	Welch Two Sample t-test

data:  bdiv_ngtax$MC4.vs.EXP and bdiv_qiime_default$MC4.vs.EXP
t = -29.03, df = 155.22, p-value < 2.2e-16
alternative hypothesis: true difference in means is not equal to 0
99 percent confidence interval:
 -0.2010843 -0.1679342
sample estimates:
 mean of x  mean of y
0.06236392 0.24687314


3. t test means beta diversity distances within MC type. NG-Tax versus QIIME default parameters.


> t.test(bdiv_ngtax$MC1.vs.MC1,bdiv_qiime_default$MC1.vs.MC1,conf.level=0.99)

	Welch Two Sample t-test

data:  bdiv_ngtax$MC1.vs.MC1 and bdiv_qiime_default$MC1.vs.MC1
t = -9.0415, df = 77.596, p-value = 9.357e-14
alternative hypothesis: true difference in means is not equal to 0
99 percent confidence interval:
 -0.1852206 -0.1014849
sample estimates:
 mean of x  mean of y
0.07036234 0.21371508

> t.test(bdiv_ngtax$MC2.vs.MC2,bdiv_qiime_default$MC2.vs.MC2,conf.level=0.99)

	Welch Two Sample t-test

data:  bdiv_ngtax$MC2.vs.MC2 and bdiv_qiime_default$MC2.vs.MC2
t = -36.358, df = 1039.6, p-value < 2.2e-16
alternative hypothesis: true difference in means is not equal to 0
99 percent confidence interval:
 -0.1482988 -0.1286425
sample estimates:
 mean of x  mean of y
0.04229213 0.18076278

> t.test(bdiv_ngtax$MC3.vs.MC3,bdiv_qiime_default$MC3.vs.MC3,conf.level=0.99)

	Welch Two Sample t-test

data:  bdiv_ngtax$MC3.vs.MC3 and bdiv_qiime_default$MC3.vs.MC3
t = -9.458, df = 70.514, p-value = 3.588e-14
alternative hypothesis: true difference in means is not equal to 0
99 percent confidence interval:
 -0.2527903 -0.1422233
sample estimates:
 mean of x  mean of y
0.04389042 0.24139724

> t.test(bdiv_ngtax$MC4.vs.MC4,bdiv_qiime_default$MC4.vs.MC4,conf.level=0.99)

	Welch Two Sample t-test

data:  bdiv_ngtax$MC4.vs.MC4 and bdiv_qiime_default$MC4.vs.MC4
t = -23.986, df = 562.86, p-value < 2.2e-16
alternative hypothesis: true difference in means is not equal to 0
99 percent confidence interval:
 -0.1837570 -0.1480085
sample estimates:
 mean of x  mean of y
0.03664946 0.20253222

4. F test variance beta diversity distances within MC type. NG-Tax versus QIIME default parameters.


> var.test(bdiv_ngtax$MC1.vs.MC1,bdiv_qiime_default$MC1.vs.MC1,conf.level=0.99)

	F test to compare two variances

data:  bdiv_ngtax$MC1.vs.MC1 and bdiv_qiime_default$MC1.vs.MC1
F = 0.03272, num df = 14, denom df = 65, p-value = 1.889e-08
alternative hypothesis: true ratio of variances is not equal to 1
99 percent confidence interval:
 0.01263664 0.11906222
sample estimates:
ratio of variances
        0.03271961

> var.test(bdiv_ngtax$MC2.vs.MC2,bdiv_qiime_default$MC2.vs.MC2,conf.level=0.99)

	F test to compare two variances

data:  bdiv_ngtax$MC2.vs.MC2 and bdiv_qiime_default$MC2.vs.MC2
F = 0.03608, num df = 209, denom df = 860, p-value < 2.2e-16
alternative hypothesis: true ratio of variances is not equal to 1
99 percent confidence interval:
 0.02750705 0.04832511
sample estimates:
ratio of variances
        0.03607978

> var.test(bdiv_ngtax$MC3.vs.MC3,bdiv_qiime_default$MC3.vs.MC3,conf.level=0.99)

	F test to compare two variances

data:  bdiv_ngtax$MC3.vs.MC3 and bdiv_qiime_default$MC3.vs.MC3
F = 0.010647, num df = 14, denom df = 65, p-value = 8.594e-12
alternative hypothesis: true ratio of variances is not equal to 1
99 percent confidence interval:
 0.00411210 0.03874414
sample estimates:
ratio of variances
        0.01064732

> var.test(bdiv_ngtax$MC4.vs.MC4,bdiv_qiime_default$MC4.vs.MC4,conf.level=0.99)

	F test to compare two variances

data:  bdiv_ngtax$MC4.vs.MC4 and bdiv_qiime_default$MC4.vs.MC4
F = 0.019521, num df = 119, denom df = 495, p-value < 2.2e-16
alternative hypothesis: true ratio of variances is not equal to 1
99 percent confidence interval:
 0.01368327 0.02889983
sample estimates:
ratio of variances
        0.01952139


5. F test variance beta diversity distances between MC type. NG-Tax versus QIIME default parameters.


> var.test(bdiv_ngtax$MC1.vs.MC2,bdiv_qiime_default$MC1.vs.MC2,conf.level=0.99)

	F test to compare two variances

data:  bdiv_ngtax$MC1.vs.MC2 and bdiv_qiime_default$MC1.vs.MC2
F = 0.059282, num df = 125, denom df = 503, p-value < 2.2e-16
alternative hypothesis: true ratio of variances is not equal to 1
99 percent confidence interval:
 0.04184057 0.08696998
sample estimates:
ratio of variances
        0.05928172

> var.test(bdiv_ngtax$MC1.vs.MC3,bdiv_qiime_default$MC1.vs.MC3,conf.level=0.99)

	F test to compare two variances

data:  bdiv_ngtax$MC1.vs.MC3 and bdiv_qiime_default$MC1.vs.MC3
F = 0.02426, num df = 35, denom df = 143, p-value < 2.2e-16
alternative hypothesis: true ratio of variances is not equal to 1
99 percent confidence interval:
 0.01284856 0.05201223
sample estimates:
ratio of variances
         0.0242604

> var.test(bdiv_ngtax$MC1.vs.MC4,bdiv_qiime_default$MC1.vs.MC4,conf.level=0.99)

	F test to compare two variances

data:  bdiv_ngtax$MC1.vs.MC4 and bdiv_qiime_default$MC1.vs.MC4
F = 0.050402, num df = 95, denom df = 383, p-value < 2.2e-16
alternative hypothesis: true ratio of variances is not equal to 1
99 percent confidence interval:
 0.03387798 0.07850973
sample estimates:
ratio of variances
        0.05040152

> var.test(bdiv_ngtax$MC2.vs.MC3,bdiv_qiime_default$MC2.vs.MC3,conf.level=0.99)

	F test to compare two variances

data:  bdiv_ngtax$MC2.vs.MC3 and bdiv_qiime_default$MC2.vs.MC3
F = 0.026409, num df = 125, denom df = 503, p-value < 2.2e-16
alternative hypothesis: true ratio of variances is not equal to 1
99 percent confidence interval:
 0.01863957 0.03874429
sample estimates:
ratio of variances
        0.02640944

> var.test(bdiv_ngtax$MC2.vs.MC4,bdiv_qiime_default$MC2.vs.MC4,conf.level=0.99)

	F test to compare two variances

data:  bdiv_ngtax$MC2.vs.MC4 and bdiv_qiime_default$MC2.vs.MC4
F = 0.027494, num df = 335, denom df = 1343, p-value < 2.2e-16
alternative hypothesis: true ratio of variances is not equal to 1
99 percent confidence interval:
 0.02214418 0.03458170
sample estimates:
ratio of variances
        0.02749449

> var.test(bdiv_ngtax$MC3.vs.MC4,bdiv_qiime_default$MC3.vs.MC4,conf.level=0.99)

	F test to compare two variances

data:  bdiv_ngtax$MC3.vs.MC4 and bdiv_qiime_default$MC3.vs.MC4
F = 0.025848, num df = 95, denom df = 383, p-value < 2.2e-16
alternative hypothesis: true ratio of variances is not equal to 1
99 percent confidence interval:
 0.01737382 0.04026255
sample estimates:
ratio of variances
        0.02584767
```
