# Supplementary material for: NG-Tax, a highly accurate and validated pipeline for analysis of 16S rRNA amplicons from complex biomes
Source: F1000Res. 2018 Nov 23;5:1791. Originally published 2016 Jul 22. [Version 2] doi: 10.12688/f1000research.9227.2 (PMC6419982; doi:10.12688/f1000research.9227.2)
Supplement: Raw data of NG-Tax pipeline for analysis of 16S rRNA amplicons from complex biome [file f1000research-5-18667-s0000.tgz › 29a888b8-2acd-4783-bd99-cd03cb48685b_Dataset_1_v2.zip!/Dataset 1/Dataset 1.3 Number of reads.docx]

Dataset 1.3: These tables show the number reads in each pipeline at specific stages in the analysis and the final number of OTUs per sample. For NG-Tax these are unique sequences, whereas in QIIME they are clustered at 97% similarity.

| **NG-Tax** | | | | |
| --- | --- | --- | --- | --- |
| **Sample name** | **Reads matching barcodes** | **Reads passing detection threshold** | **Reads after error correction** | **Number of OTUs**  **(Non clustered)** |
| Mc.1.1.l01 | 187028 | 104282 | 135946 | 76 |
| Mc.1.1.l02 | 477491 | 240965 | 335822 | 49 |
| Mc.1.2.l01 | 111121 | 62624 | 80994 | 76 |
| Mc.1.2.l02 | 382804 | 183556 | 248173 | 44 |
| Mc.1.3.l01 | 96658 | 52470 | 68520 | 71 |
| Mc.1.3.l02 | 448432 | 214529 | 304306 | 52 |
| Mc.2.1.l01 | 19191 | 6440 | 9462 | 126 |
| Mc.2.1.l02 | 261866 | 135245 | 182941 | 64 |
| Mc.2.1.l03 | 38520 | 27983 | 35609 | 57 |
| Mc.2.1.l04 | 215171 | 153897 | 196437 | 59 |
| Mc.2.1.l05 | 114637 | 82104 | 104610 | 63 |
| Mc.2.1.l06 | 111719 | 80140 | 102023 | 60 |
| Mc.2.1.l07 | 140255 | 98449 | 126226 | 60 |
| Mc.2.2.l01 | 30994 | 15528 | 21901 | 123 |
| Mc.2.2.l02 | 326248 | 157460 | 228950 | 64 |
| Mc.2.2.100v.dup.l02 | 172480 | 83685 | 110801 | 61 |
| Mc.2.2.100v.l02 | 363877 | 181603 | 241254 | 64 |
| Mc.2.2.30cy.l02 | 190814 | 79050 | 107859 | 65 |
| Mc.2.2.35cy.l02 | 92342 | 35016 | 47897 | 73 |
| Mc.2.2.dup.l02 | 362247 | 170960 | 232169 | 64 |
| Mc.2.2.l03 | 59881 | 43328 | 55164 | 60 |
| Mc.2.2.l04 | 84260 | 60823 | 77705 | 59 |
| Mc.2.2.l05 | 54971 | 39703 | 50609 | 62 |
| Mc.2.2.l06 | 50464 | 36481 | 46469 | 62 |
| Mc.2.2.l07 | 35646 | 25441 | 32379 | 59 |
| Mc.2.3.l01 | 10267 | 5197 | 7220 | 117 |
| Mc.2.3.l02 | 405154 | 200388 | 291659 | 68 |
| Mc.3.1.l01 | 5090 | 2641 | 3608 | 142 |
| Mc.3.1.l02 | 168144 | 79434 | 115527 | 73 |
| Mc.3.2.l01 | 3089 | 1869 | 2363 | 141 |
| Mc.3.2.l02 | 282229 | 147953 | 208602 | 76 |
| Mc.3.3.l01 | 9449 | 5144 | 6932 | 111 |
| Mc.3.3.l02 | 149705 | 73957 | 105862 | 69 |
| Mc.4.1.l01 | 6100 | 2107 | 3028 | 121 |
| Mc.4.1.l02 | 111474 | 58098 | 82877 | 59 |
| Mc.4.1.l03 | 35747 | 23358 | 33466 | 67 |
| Mc.4.1.l04 | 60791 | 39171 | 56796 | 65 |
| Mc.4.1.l05 | 41362 | 27363 | 38897 | 73 |
| Mc.4.1.l06 | 34381 | 22780 | 32472 | 72 |
| Mc.4.1.l07 | 25605 | 16829 | 23890 | 80 |
| Mc.4.2.l01 | 4017 | 1907 | 2673 | 101 |
| Mc.4.2.l02 | 120115 | 60725 | 86069 | 54 |
| Mc.4.2.l03 | 113217 | 73641 | 105495 | 70 |
| Mc.4.2.l04 | 141078 | 90856 | 131257 | 65 |
| Mc.4.2.l05 | 94804 | 61990 | 88354 | 70 |
| Mc.4.2.l06 | 95338 | 62823 | 89268 | 74 |
| Mc.4.2.l07 | 73984 | 47862 | 68593 | 76 |
| Mc.4.3.l01 | 8991 | 4270 | 6054 | 113 |
| Mc.4.3.l02 | 441365 | 214673 | 309942 | 53 |

| **QIIME** | | | |
| --- | --- | --- | --- |
| **Sample name** | **Reads before filtering** | **Reads after filtering** | **Number of OTUs (clustered at 97% similarity)** |
| Mc.1.1.l01.1 | 425816 | 376610 | 458 |
| Mc.1.1.l01.2 | 396262 | 357049 | 459 |
| Mc.1.1.l02.1 | 520542 | 498049 | 510 |
| Mc.1.1.l02.2 | 557648 | 536823 | 511 |
| Mc.1.2.l01.1 | 272337 | 244735 | 438 |
| Mc.1.2.l01.2 | 267379 | 243698 | 432 |
| Mc.1.2.l02.1 | 415656 | 397290 | 505 |
| Mc.1.2.l02.2 | 437983 | 420936 | 501 |
| Mc.1.3.l01.1 | 240253 | 214204 | 432 |
| Mc.1.3.l01.2 | 240957 | 218301 | 435 |
| Mc.1.3.l02.1 | 481567 | 459998 | 506 |
| Mc.1.3.l02.2 | 518989 | 498625 | 513 |
| Mc.2.1.l01.1 | 71892 | 62214 | 409 |
| Mc.2.1.l01.2 | 76969 | 67523 | 431 |
| Mc.2.1.l02.1 | 306438 | 293960 | 527 |
| Mc.2.1.l02.2 | 327146 | 315656 | 536 |
| Mc.2.1.l03.1 | 151213 | 128221 | 586 |
| Mc.2.1.l03.2 | 123464 | 109979 | 579 |
| Mc.2.1.l04.1 | 416636 | 370241 | 675 |
| Mc.2.1.l04.2 | 381940 | 351658 | 671 |
| Mc.2.1.l05.1 | 269394 | 249071 | 611 |
| Mc.2.1.l05.2 | 223032 | 210605 | 597 |
| Mc.2.1.l06.1 | 264449 | 247199 | 625 |
| Mc.2.1.l06.2 | 221179 | 209874 | 599 |
| Mc.2.1.l07.1 | 280000 | 257186 | 634 |
| Mc.2.1.l07.2 | 258445 | 242587 | 620 |
| Mc.2.2.100v.dup.l02.1 | 215668 | 207471 | 502 |
| Mc.2.2.100v.dup.l02.2 | 214169 | 206731 | 506 |
| Mc.2.2.100v.l02.1 | 468984 | 449223 | 555 |
| Mc.2.2.100v.l02.2 | 483800 | 465432 | 548 |
| Mc.2.2.30cy.l02.1 | 240998 | 231052 | 530 |
| Mc.2.2.30cy.l02.2 | 238470 | 229577 | 520 |
| Mc.2.2.35cy.l02.1 | 130285 | 124010 | 502 |
| Mc.2.2.35cy.l02.2 | 128477 | 121792 | 496 |
| Mc.2.2.dup.l02.1 | 403711 | 387912 | 550 |
| Mc.2.2.dup.l02.2 | 418262 | 403179 | 548 |
| Mc.2.2.l01.1 | 116048 | 101506 | 432 |
| Mc.2.2.l01.2 | 114349 | 101893 | 425 |
| Mc.2.2.l02.1 | 377533 | 362415 | 554 |
| Mc.2.2.l02.2 | 397332 | 383412 | 549 |
| Mc.2.2.l03.1 | 196230 | 171960 | 597 |
| Mc.2.2.l03.2 | 148309 | 133918 | 577 |
| Mc.2.2.l04.1 | 238445 | 216430 | 660 |
| Mc.2.2.l04.2 | 189956 | 176579 | 631 |
| Mc.2.2.l05.1 | 184922 | 172620 | 579 |
| Mc.2.2.l05.2 | 130202 | 123123 | 549 |
| Mc.2.2.l06.1 | 169764 | 159670 | 580 |
| Mc.2.2.l06.2 | 126228 | 119639 | 559 |
| Mc.2.2.l07.1 | 107576 | 99678 | 580 |
| Mc.2.2.l07.2 | 86686 | 81716 | 541 |
| Mc.2.3.l01.1 | 51123 | 44713 | 406 |
| Mc.2.3.l01.2 | 52437 | 46984 | 402 |
| Mc.2.3.l02.1 | 497164 | 477006 | 563 |
| Mc.2.3.l02.2 | 523196 | 504027 | 561 |
| Mc.3.1.l01.1 | 32996 | 28394 | 402 |
| Mc.3.1.l01.2 | 39871 | 34884 | 398 |
| Mc.3.1.l02.1 | 222300 | 212558 | 524 |
| Mc.3.1.l02.2 | 229117 | 219969 | 522 |
| Mc.3.2.l01.1 | 22683 | 19779 | 375 |
| Mc.3.2.l01.2 | 25094 | 22203 | 380 |
| Mc.3.2.l02.1 | 343344 | 330237 | 529 |
| Mc.3.2.l02.2 | 358367 | 345996 | 534 |
| Mc.3.3.l01.1 | 57874 | 49771 | 405 |
| Mc.3.3.l01.2 | 54209 | 47603 | 402 |
| Mc.3.3.l02.1 | 199978 | 191912 | 523 |
| Mc.3.3.l02.2 | 203985 | 196705 | 521 |
| Mc.4.1.l01.1 | 35256 | 30748 | 386 |
| Mc.4.1.l01.2 | 34216 | 30321 | 390 |
| Mc.4.1.l02.1 | 156096 | 150615 | 464 |
| Mc.4.1.l02.2 | 149507 | 144806 | 468 |
| Mc.4.1.l03.1 | 113346 | 101826 | 507 |
| Mc.4.1.l03.2 | 81402 | 74335 | 498 |
| Mc.4.1.l04.1 | 153187 | 141187 | 581 |
| Mc.4.1.l04.2 | 126180 | 118447 | 559 |
| Mc.4.1.l05.1 | 124147 | 116878 | 504 |
| Mc.4.1.l05.2 | 84678 | 80826 | 492 |
| Mc.4.1.l06.1 | 102672 | 97558 | 502 |
| Mc.4.1.l06.2 | 75289 | 72092 | 474 |
| Mc.4.1.l07.1 | 69919 | 66165 | 490 |
| Mc.4.1.l07.2 | 57916 | 55292 | 473 |
| Mc.4.2.l01.1 | 42830 | 36159 | 394 |
| Mc.4.2.l01.2 | 46354 | 40045 | 398 |
| Mc.4.2.l02.1 | 164697 | 158678 | 476 |
| Mc.4.2.l02.2 | 168357 | 162758 | 486 |
| Mc.4.2.l03.1 | 254749 | 226995 | 554 |
| Mc.4.2.l03.2 | 218426 | 199909 | 539 |
| Mc.4.2.l04.1 | 282814 | 259243 | 602 |
| Mc.4.2.l04.2 | 257360 | 241241 | 587 |
| Mc.4.2.l05.1 | 219540 | 206689 | 549 |
| Mc.4.2.l05.2 | 175414 | 167458 | 519 |
| Mc.4.2.l06.1 | 217126 | 205574 | 562 |
| Mc.4.2.l06.2 | 184720 | 176261 | 522 |
| Mc.4.2.l07.1 | 146126 | 137857 | 547 |
| Mc.4.2.l07.2 | 133822 | 127731 | 527 |
| Mc.4.3.l01.1 | 70310 | 60311 | 403 |
| Mc.4.3.l01.2 | 72651 | 63543 | 410 |
| Mc.4.3.l02.1 | 484320 | 465412 | 531 |
| Mc.4.3.l02.2 | 514732 | 497906 | 527 |
