# Supplementary material for: NG-Tax, a highly accurate and validated pipeline for analysis of 16S rRNA amplicons from complex biomes
Source: F1000Res. 2018 Nov 23;5:1791. Originally published 2016 Jul 22. [Version 2] doi: 10.12688/f1000research.9227.2 (PMC6419982; doi:10.12688/f1000research.9227.2)
Supplement: Raw data of NG-Tax pipeline for analysis of 16S rRNA amplicons from complex biome [file f1000research-5-18667-s0000.tgz › 29a888b8-2acd-4783-bd99-cd03cb48685b_Dataset_1_v2.zip!/Dataset 1/Dataset 1.5 Code employed in the analysis.docx]

**Code employed in the analysis.**

**NG-Tax code.**

1. **Build libraries**

\# Download sample files for ENA repository (accession number PRJEB11702, decompress them and concatenate them to build the different libraries.

cat *l01_1.fastq > lib01_1.fastq

cat *l01_2.fastq > lib01_2.fastq

cat *l02_1.fastq > lib02_1.fastq

cat *l02_2.fastq > lib02_2.fastq

cat *l03_1.fastq > lib03_1.fastq

cat *l03_2.fastq > lib03_2.fastq

cat *l04_1.fastq > lib04_1.fastq

cat *l04_2.fastq > lib04_2.fastq

cat *l05_1.fastq > lib05_1.fastq

cat *l05_2.fastq > lib05_2.fastq

cat *l06_1.fastq > lib06_1.fastq

cat *l06_2.fastq > lib06_2.fastq

cat *l07_1.fastq > lib07_1.fastq

cat *l07_2.fastq > lib07_2.fastq

**2. Create customized 16S databases**

**2.1 Customized V5-V6 database**

customized_database_generator -d Silva_111_full_unique.fasta -t SILVA_db/tax_slv_ssu_128.txt -x Silva_taxonomic_table_v56 -k [AG]GGATTAGATACCC -p CGAC[AG][AG]CCATGCA[ACGT]CACCT -q AGGTG[ACGT]TGCATGG[TC][TC]GTCG -f primer_BSF784_76_nt_1mm_db -r primer_R1064_71_nt_1mm_db -o 76 -e 71 -y primer_BSF784_1mm -z primer_R1064_1mm

**2.2 Customized V4 database**

customized_database_generator -d Silva_111_full_unique.fasta -t SILVA_db/tax_slv_ssu_128.txt -x Silva_taxonomic_table_v4 -k GTGCCAGC[AC]GCCGCGGTAA -p GGACTAC[ACT][ACG]GGGT[AT]TCTAAT -q ATTAGA[AT]ACCC[TCG][ATG]GTAGTCC -f primer_F515_71_nt_1mm_db -r primer_R806_70_nt_1mm_db -o 71 -e 70 -y primer_F515_1mm -z primer_R806_1mm

**3. Filtering libraries 01 to 07.**

library_filtering -a lib01_1.fastq -b lib01_2.fastq -p Mock_communities -n 01 -f [AG]GGATTAGATACCC -r CGAC[AG][AG]CCATGCA[ACGT]CACCT -l 8

library_filtering -a lib02_1.fastq -b lib02_2.fastq -p Mock_communities -n 02 -f GTGCCAGC[AC]GCCGCGGTAA -r GGACTAC[ACT][ACG]GGGT[AT]TCTAAT -l 8

library_filtering.sh -a lib03_1.fastq -b lib03_2.fastq -p Mock_communities -n 03 -f GTGCCAGC[AC]GCCGCGGTAA -r GGACTAC[ACT][ACG]GGGT[AT]TCTAAT -l 8

library_filtering.sh -a lib04_1.fastq -b lib04_2.fastq -p Mock_communities -n 04 -f GTGCCAGC[AC]GCCGCGGTAA -r GGACTAC[ACT][ACG]GGGT[AT]TCTAAT -l 8

library_filtering.sh -a lib05_1.fastq -b lib05_2.fastq -p Mock_communities -n 05 -f GTGCCAGC[AC]GCCGCGGTAA -r GGACTAC[ACT][ACG]GGGT[AT]TCTAAT -l 8

library_filtering.sh -a lib06_1.fastq -b lib06_2.fastq -p Mock_communities -n 06 -f GTGCCAGC[AC]GCCGCGGTAA -r GGACTAC[ACT][ACG]GGGT[AT]TCTAAT -l 8

library_filtering.sh -a lib07_1.fastq -b lib07_2.fastq -p Mock_communities -n 07 -f GTGCCAGC[AC]GCCGCGGTAA -r GGACTAC[ACT][ACG]GGGT[AT]TCTAAT -l 8

**4. OTU picking**

**4.1 OTU picking V5V6 samples.**

otu_picking_pair_end_read.sh -m Mock_communities_V5V6.txt -p Mock_communities -a 0.1 -c 0.985 -f primer_BSF784_76_nt_1mm_db -r primer_R1064_71_nt_1mm_db -o 76 -e 71 -t Silva_taxonomic_table_v56 -q 2 -k 100 -n 24

**4.2 OTU picking V4 samples.**

otu_picking_pair_end_read.sh -m Mock_communities_V4.txt -p Mock_communities -a 0.1 -c 0.985 -f primer_F515_71_nt_1mm_db -r primer_R806_70_nt_1_mm_db -o 71 -e 70 -t Silva_taxonomic_table_v4 txt -q 2 -k 100 -n 24

**5. OTU recovery for reassignment or removal.**

**5.1 OTU recovery for reassignment or removal for V5V6 samples.**

otu_recovery_by_pattern.sh -t tax_files -n non_assigned_V5V6 -p NA -s taxonomy

otu_recovery_by_pattern.sh -t tax_files -n Parabacteroides_V5V6 -p Porphyromonadaceae -s taxonomy

**# 3 otus from V5V6 samples (containing only 12 reads in total) could not be assigned. They were blasted against the NCBI NR repository and the hits obtained did not allow to reclassify the OTUs, therefore they were considered artefacts and removed.**

**# Files Mock_communities_V5V6_non_assigned_alternative_taxonomy_file and Mock_communities_V5V6_Parabacteroides_alternative_taxonomy_file were concatenated.**

cat otu_retrievement_files/non_assigned_V5V6_alternative_taxonomy_file otu_retrievement_files/Parabacteroides_V5V6_alternative_taxonomy_file > otu_retrievement_files/Mock_communities_V5V6_alternative_taxonomy_file

**5.2 OTU recovery for reassignment or removal for V4 samples.**

**# Recover Parabacteroides reads from V4 samples.**

otu_recovery_by_pattern.sh -t tax_files -n Parabacteroides_V4 -p TACGGAGGATCCGAGCGTTATCCGGATTTATTGGGTTTAAAGGGTGCGTAGGCGGCCTTTTAAGTCAGCGGCCTGTTTGATCCCCACGCTTTCG -s sequence

**6. Taxonomical reassignments**

**# Removal of Parabacteroides and NA OTUs from V5V6 samples. The word remove was written in the 3^rd^ column of Mock_communities_V56_alternative_taxonomy_file**

taxonomical_reassignment.sh -a otu_retrievement_files/ Mock_communities_V5V6_alternative_taxonomy_file -t tax_files

**# Removal of Parabacteroides OTUs from V4 samples. The word remove was written in the 3^rd^ column of Mock_communities_V4_alternative_Parabacteroide_taxonomy_file**

taxonomical_reassignment.sh -a out_retrievement_files/Parabacteroides_V4_alternative_taxonomy_file -t tax_files

**7. Add theoretical tax files.**

**# Theoretical tax files for Mc.1.t.l44, Mc.1.t.l56, Mc.2.t.l56, Mc.2.t.l44, Mc.3.t.l44, Mc.3.t.l56, Mc.4.t.l44 and Mc.4.t.l56 are added to the folder alternative_reassigned_tax_files for their respective region.**

**8. Region comparison**

**8.1 Region comparison V4 samples.**

region_16S_comparator.sh -m Mock_communities_V4_plus_theoretical.txt -t alternative_reassigned_tax_files -f primer_BSF784_76_nt_1mm_db -r primer_R1064_71_nt_1mm_db -p 1 &

**8.2 Region comparison V5V6 samples.**

region_16S_comparator.sh -m Mock_communities_V5V6_plus_theorical.txt -t alternative_reassigned_tax_files -f primer_F515_71_nt_1mm_db -r primer_R806_70_nt_1mm_db -p 2 &

**9. Make biom file.**

**# Samples for both regions are copied to the folder both_regions_tax_files.**

make_biom_file.sh -m Mock_communities.txt -t both_regions_tax_files

**10. Make dnd tree file using uclust with Mock_communities_otu_database.fa**

**11. Downstream analysis with QIIME.**

biom summarize-table -i Mock_communities.biom -o sample_size_mock_communities

summarize_taxa_through_plots.py -i Mock_communities.biom -o taxa_summary_mc

echo "alpha_diversity:metrics shannon,simpson,PD_whole_tree,chao1,observed_species" > alpha_params.txt

echo "beta_diversity:metrics unweighted_unifrac,weighted_unifrac,bray_curtis" > beta_params.txt

alpha_rarefaction.py -i Mock_communities.biom -m Mock_communities.txt -o alpha_rarefaction_1911_mc -t Mock_communities_otu_database.dnd -p alpha_params.txt -e 1911

beta_diversity_through_plots.py -i Mock_communities.biom -m Mock_communities.txt -o beta_diversity_mc -t Mock_communities_otu_database.dnd -p beta_params.txt

jackknifed_beta_diversity.py -i Mock_communities.biom -t Mock_communities_otu_database.dnd -m Mock_communities.txt -o jack_beta_diversity_1911_mc -e 1911 -p beta_params.txt

**QIIME Code**

1. **Extract the barcodes. For each library.**

extract_barcodes.py -f library_3_1.fastq -c barcode_single_end -m mapping_file_forQIIME_comparison_MC_lib1.1.txt -l 8 -o processed_seqs1.1 -a

2. **Extract the linker. For each library.**

extract_barcodes.py -f processed_seqs1.1/reads.fastq -c barcode_single_end -m mapping_file_forQIIME_comparison_MC_lib1.1.txt -l 2 -o processed_seqs1.1/wo_linker -a

3. **Demultiplex using the reads without linker and the 8nt barcode. For each library.**

split_libraries_fastq.py -i processed_seqs1.1/wo_linker/reads.fastq -o Split_samples1.1/wo_linker/ -b processed_seqs1.1/barcodes.fastq -m mapping_file_forQIIME_comparison_MC_lib1.1.txt --barcode_type 8

4. **Pick closed reference OTUs against the default GreenGeengenes database using the "pick_otus:enable_rev_strand_match True" line in the parameter file. For each library.**

pick_closed_reference_otus.py -i Split_samples1.1/wo_linker/seqs.fna -o otus/otus1.1/wo_linker/ -p params_OTU_picking.txt -a -O 16

5. **Merged all resulting OTU tables**

merge_otu_tables.py -i otu_table1.1.biom,otu_table2.1.biom,otu_table3.1.biom,otu_table4.1.biom,otu_table5.1.biom,otu_table6.1.biom,otu_table7.1.biom,otu_table1.2.biom,otu_table2.2.biom,otu_table3.2.biom,otu_table4.2.biom,otu_table5.2.biom,otu_table6.2.biom,otu_table7.2.biom -o merged_otu_table_wo_linker.biom

6. **Apply advised default filtering parameters**

filter_otus_from_otu_table.py -i ~/qiime/otu_tables/wo_linker/merged_otu_table_wo_linker.biom -o ~/qiime/wo_linker/merged_otu_table_filtered_wo_linker.biom --min_count_fraction 0.00005

7. **Due to the quality issues with the Parabacteroides sequences these (family: Porphyromonadaceae) were filtered out manually because the normal command does not work with special characters.**

filter_taxa_from_otu_table.py -i ~/qiime/wo_linker/merged_otu_table_filtered_wo_linker.biom -o ~/qiime/wo_linker/otu_table_no_Porphyromonadaceae.biom -n k__Bacteria;p__Bacteroidetes;c__Bacteroidia;o__Bacteroidales;f__Porphyromonadaceae

8. **Manual removal of k__Bacteria;p__Bacteroidetes;c__Bacteroidia;o__Bacteroidales;f__Porphyromonadaceae**

biom convert -i ~/qiime/wo_linker/merged_otu_table_filtered_wo_linker.biom -o ~/qiime/wo_linker/merged_otu_table.filtered.txt --to-tsv --header-key taxonomy

biom convert -i ~/qiime/wo_linker/merged_otu_table.filtered.txt -o ~/qiime/wo_linker/otu_table_no_Porphyromonadaceae.biom --to-hdf5 --table-type="OTU table" --process-obs-metadata taxonomy

9. **OTU picking for the theoretical sequences**

pick_closed_reference_otus.py -i ~/qiime/wo_linker/mc_theoretical_for_qiime/Mc.1.t.lfV56.fna -o ~/qiime/wo_linker/full_length_reference/1.fV56 -p ~/qiime/params_OTU_picking.txt -a -O 16

10. **Merge all theoretical OTU tables**

merge_otu_tables.py -i qiime/wo_linker/full_length_reference/1.fV56/otu_table.biom,qiime/wo_linker/full_length_reference/2.fV56/otu_table.biom,qiime/wo_linker/full_length_reference/3.fV56/otu_table.biom,qiime/wo_linker/full_length_reference/4.fV56/otu_table.biom,qiime/wo_linker/full_length_reference/1.fV44/otu_table.biom,qiime/wo_linker/full_length_reference/2.fV44/otu_table.biom,qiime/wo_linker/full_length_reference/3.fV44/otu_table.biom,qiime/wo_linker/full_length_reference/4.fV44/otu_table.biom,qiime/wo_linker/full_length_reference/1.rV56/otu_table.biom,qiime/wo_linker/full_length_reference/2.rV56/otu_table.biom,qiime/wo_linker/full_length_reference/3.rV56/otu_table.biom,qiime/wo_linker/full_length_reference/4.rV56/otu_table.biom,qiime/wo_linker/full_length_reference/1.rV44/otu_table.biom,qiime/wo_linker/full_length_reference/2.rV44/otu_table.biom,qiime/wo_linker/full_length_reference/3.rV44/otu_table.biom,qiime/wo_linker/full_length_reference/4.rV44/otu_table.biom -o ~/qiime/wo_linker/merged_reference.biom

11. **Merge the sequenced and theoretical data for analysis**

merge_otu_tables.py –i ~/qiime/wo_linker/merged_reference.biom,qiime/wo_linker/otu_table_no_Porphyromonadaceae.biom –o ~/qiime/wo_linker/Reference_and_sequenced_data.biom

12. **Downstream analysis.**

summarize_taxa_through_plots.py -i ~/qiime/wo_linker/Reference_and_sequenced_data.biom -o taxa_summary_qiime_mc

beta_diversity_through_plots.py -i ~/qiime/wo_linker/Reference_and_sequenced_data.biom -m ~/qiime/mapping_all.txt -a -O 16 -e 10000 -t/usr/local/lib/python2.7/dist-packages/qiime_default_reference/gg_13_8_otus/trees/97_otus.tree -o ~/qiime/wo_linker/beta_div_even10000_reference_and_sequenced_data -f

alpha_rarefaction.py -i ~/qiime/wo_linker/Reference_and_sequenced_data.biom -n 30 -e 20000 -o ~/qiime/wo_linker/a_rare/all_no_porphyr_with_reference -m ~/qiime/mapping_all.txt -t /usr/local/lib/python2.7/dist-packages/qiime_default_reference/gg_13_8_otus/trees/97_otus.tree -f -a -O 16 -p ~/qiime/params_alpha_diversity.txt
