# Supplementary material for: NG-Tax, a highly accurate and validated pipeline for analysis of 16S rRNA amplicons from complex biomes
Source: F1000Res. 2018 Nov 23;5:1791. Originally published 2016 Jul 22. [Version 2] doi: 10.12688/f1000research.9227.2 (PMC6419982; doi:10.12688/f1000research.9227.2)
Supplement: Raw data of NG-Tax pipeline for analysis of 16S rRNA amplicons from complex biome [file f1000research-5-18667-s0000.tgz › 29a888b8-2acd-4783-bd99-cd03cb48685b_Dataset_1_v2.zip!/Dataset 1/Dataset 1.13 NG-tax user manual.zip!/Dataset 1.13 NG-tax user manual/NG-Tax user manual.html]

NG-Tax/NG-Tax-v1 user manual.md at master · JavierRamiroGarcia/NG-Tax · GitHub


Skip to content


- Features
- Business
- Explore
- Marketplace
- Pricing

- In this repository

  All GitHub
  ↵

  Jump to
  ↵

- No suggested jump to results

Sign in
or
Sign up

- Watch

  1
- Star

  0
- Fork

  0

# JavierRamiroGarcia/**NG-Tax**

Code

 


Issues
0

 


Pull requests
0

 


Projects
0


Insights

Permalink


Dismiss

### Join GitHub today

GitHub is home to over 28 million
developers working together to host and review code, manage projects,
and build software together.

Sign up

*Branch:*
master

Switch branches/tags

- Branches
- Tags

master

Nothing to show

Nothing to show

Find file

Copy path

NG-Tax/NG-Tax-v1/**NG-Tax-v1 user manual.md**

7f161b7
2 minutes ago

JavierRamiroGarcia
add version of USEARCH

**1** contributor


### Users who have contributed to this file

- JavierRamiroGarcia

Raw
Blame
History

executable file

484 lines (291 sloc)
18.1 KB

NG-Tax user manual.  
Nov 2018, version 1.0  
Last version available at https://github.com/JavierRamiroGarcia/NG-Tax.git

# 1. Introduction.

NG-Tax, a pipeline for 16S rRNA amplicon sequencing analysis of complex biomes using NGS data.
It was validated with four different Mock Communities, specifically designed to tackle issues
regarding optimization of routinely used filtering parameters. It was designed for short paired-end
reads that are translated to a biom file that can be used in QIIME and other available pipelines for
downstream analysis. NG-Tax presents high robustness against technical bias associated with 16S
rRNA gene amplicon studies that improves comparability between studies and facilitates efforts
towards standardization.

# 2. Installation Instructions.

Open a Linux terminal, move to the folder in which you want to install NG-Tax.

Getting the source code:

Download NG-Tax from GitHub by giving the following command:

```
git clone https://github.com/JavierRamiroGarcia/NG-Tax.git
```

Go to http://www.drive5.com/usearch/download.html and download the USEARCH linux version (usearch v8.1.1861\_i86linux32).
Make a copy of the file with the name usearch and place it in the bin folder of NG-Tax-v1.

Downloading the database:

To install the pipeline and clustalw, run the NG-Tax\_installer\_unix.sh script from the folder NG-Tax-v1.
It will also download the Silva database.

```
cd NG-Tax/NG-Tax-v1/
./NG-Tax_installer_unix.sh
```

The pipeline can be tested by running the script runTest.sh present in the folder named test\_set.
Run it from the test\_set folder by typing:

```
cd test_set
./runTest.sh
```

Note: This is to check whether the installation has been done correctly.

# 3. How to run NG-Tax

Create a folder for the project where you place your raw data libraries that are already decompressed

## 3.1. Create a mapping file

Create a mapping file with extension .txt containing the information for all the samples in the
project (no matter if they are in different libraries) and place it in your project folder.
The mapping file and any other file needed should be created on Linux environment to
avoid incompatibilities or should be saved in UTF-8 format.

**Your mapping file should contain these tab separated columns:**

- First column **#SampleID** name. Underscores are not allowed, but periods are allowed.  
  Every sample should have a different name.
- Second column **BarcodeSequence**, barcode associated to the sample.
- Third column **LibraryNumber**, library associated to the sample (given by the user), should contain
  two digits that can go from 01 to 99. Hence, up to 99 libraries can be included in a study.
- Fourth column **Direction**, p is the option for paired-end reads.
- Fifth column **LibraryName**, names given by the sequencing provider.
- Sixth column **ProjectName**, name given to the study.
- Last column **Description**, same name as First column.
- Between the sixth and the last column any number of metadata columns can be included.

**Your mapping file should contain these lines:**

- First line, starting by # and containing the name of the variables.
- Last line, empty line.
- Between the first and the last line, any number of samples can be included.

**Example mapping file:**

| #SampleID | BarcodeSequence | LibraryNumber | Direction | LibraryName | ProjectName | Area | Sex | Age | Description |
| --- | --- | --- | --- | --- | --- | --- | --- | --- | --- |
| Tala.17 | CTGGATAA | 01 | p | lib1\_1.fastq,lib1\_2.fastq | Mock | Talarrubias | M | Adult | Tala.17 |
| Tala.22 | ATAAGGTC | 01 | p | lib1\_1.fastq,lib1\_2.fastq | Mock | Talarrubias | M | Adult | Tala.22 |
| Trigue.6 | AATAAGGA | 01 | p | lib1\_1.fastq,lib1\_2.fastq | Mock | Trigueros | M | Adult | Trigue.6 |
| Tala.19 | TACTTATC | 02 | p | lib1\_1.fastq,lib1\_2.fastq | Mock | Talarrubias | F | Young | Tala.19 |
| Trigue.20 | ATCTCAGT | 02 | p | lib1\_1.fastq,lib1\_2.fastq | Mock | Trigueros | M | Adult | Trigue.20 |
| (empty line) |  |  |  |  |  |  |  |  |  |

## 3.2. Create customized 16S rRNA databases for your primers.

This script generates the customized databases adapted to NG-Tax by in-silico PCR, using the
primer sequences and the read length introduced by the user.
Degenerate positions can be included between brackets.
It also allows the inclusion of primer mismatches. It uses the databases
from the Silva repository downloaded during the installation.

Example:

```
customized_database_generator.sh -d SILVA_db/SILVA_128_SSUREF_tax_silva.fasta -t SILVA_db/tax_slv_ssu_128.txt -x Silva_taxonomic_table -k GTGCCAGC[AC]GCCGCGGTAA -p GGACTAC[ACT][ACG]GGGT[AT]TCTAAT -q ATTAGA[AT]ACCC[TCG][ATG]GTAGTCC -f primer_F515_71nt_1mm_db -r primer_R806_70nt_1mm_db -o 71 -e 70 -y primer_F515_1mm -z primer_R806_1mm
```

**Input:**

**-d** SILVA\_reference16S database in fasta format → SILVA\_128\_SSUREF\_tax\_silva.fasta  
**-t** taxonomy file from Silva taxonomy → slv\_ssu\_128.txt  
**-x** customized taxonomic table → Silva\_taxonomic\_table  
**-k** forward\_primer\_sequence → GTGCCAGC[AC]GCCGCGGTAA  
**-p** reverse\_primer\_sequence → GGACTAC[ACT][ACG]GGGT[AT]TCTAAT  
**-q** complementary\_reversed\_reverse\_primer\_sequence → ATTAGA[AT]ACCC[TCG][ATG]GTAGTCC  
**-f** forward\_primer\_database\_name → primer\_F515\_71nt\_1mm\_db  
**-r** reverse\_primer\_database\_name → primer\_R806\_70nt\_1mm\_db  
**-o** length\_forward\_read → 71  
**-e** length\_reverse\_read → 70

**Optional input:**

**-y** file with allowed forward primers → primer\_F515\_1mm  
**-z** file with allowed reverse primers → primer\_R806\_1mm

**Output:**

- A database with all sequences that have matching forward primer → primer\_F515\_71nt\_1mm\_db
- A database with all sequences that have matching reverse primer → primer\_R806\_70nt\_1mm\_db
- A taxonomy file that can be used in NG-Tax → taxonomy\_NG\_Tax

## 3.3. Library filtering.

This script filters those reads that don’t have matching barcodes and formats the fastq files in order
to be used in NG-Tax.  
Samples should have forward and reverse barcoded reads using the same
barcode.  
Last nucleotide of every read is also removed for quality reasons.

```
library_filtering.sh -a lib1_1.fastq -b lib1_2.fastq -p Mock -n 01 -f GTGCCAGC[AC]GCCGCGGTAA -r GGACTAC[ACT][ACG]GGGT[AT]TCTAAT -l 8
```

**Input:**

**-a** Address of the Illumina library 1 → lib1\_1.fastq  
**-b** Address of the Illumina library 2 → lib1\_2.fastq  
**-p** Name given to the project (indicated in the sixth column of the mapping file, **ProjectName**) → Mock  
**-n** number given to the library (indicated in the third column of the mapping file, **LibraryNumber**) → 01  
**-f** sequence of the forward primer, degenerate positions between brackets → GTGCCAGC[AC]GCCGCGGTAA  
**-r** sequence of the reverse primer, degenerate positions between brackets → GGACTAC[ACT][ACG]GGGT[AT]TCTAAT  
**-l** barcode nucleotide length → 8

**Output:**

- A filtered library → Mock\_01

## 3.4. OTU picking, chimera removal and taxonomic assignment.

This script demultiplexes the raw data into samples using the information contained in the mapping file.  
It also generates an OTU table per sample after removing chimeras and assigns taxonomy to the OTUs.  
NG-Tax is designed for short reads, 70-100 nucleotides is the recommended read length.  
Reads can be trimmed to this length by the script. Longer length can be
selected by the user but comparison with 70-100 nucleotide analysis is
advisable.

**OTU picking:**

Reads are ranked by abundance and OTUs are added to an OTU table for that sample
starting from the most abundant sequence until the abundance is lower than a percentage defined
by the user (the recommended threshold is 0.1).  
Then discarded reads are clustered to the OTU table allowing one mismatch.

**Chimera removal:**

OTUs are subjected to non-reference based chimera checking according to the
following principle: given three OTUs named A, B and C, C will be considered a chimera when the
following conditions are satisfied: C and A 5’ reads are identical, C and B 3’ reads are identical and
both OTUs, A and B, are at least twice as abundant as OTU C.

**Taxonomic assignment:**

For each OTU, USEARCH is used to retrieve hits of forward and reverse reads against their respective trimmed reference database.  
Then the hits that are in common between both reads are divided in 6 identity thresholds 100, 98, 97, 95, 92, 90.  
A hit belong to a certain level, for example 97, only if both reads has at least a 97 percentage identity with that hit.
Using the highest available identity threshold NG-Tax assigns the consensus taxonomy to the OTU
if this taxonomy is supported for at least half of the hits.  
Genus, Family or Order remains unassigned if the maximum identity percentage level is lower or
equal to 97%, 95% and 92% respectively.

```
OTU_picking_pair_end_read.sh -m map_Mock_communities.txt -p Mock -a 0.1 -c 0.985 -f primer_F515_71nt_1mm_db -r primer_R806_70nt_1_mm_db -o 71 -e 70 -t Silva_taxonomic_table -q 2 -k 100 -n 24
```

**Input:**

**-m** mapping file address → map\_Mock\_communities.txt  
**-p** library name (matching the ProjectName). If needed add path to the libraries → Mock  
**-a** minimum threshold detectable, expressed in percentage (0.1 recommended) → 0.1  
**-c** error correction clustering percentage (only one mismatch recommended, 0.985 for ~70 nt) →0.985  
**-f** forward primer database address (database adapted to the read length) → primer\_F515\_71nt\_1mm\_db  
**-r** reverse primer database address (database adapted to the read length) → primer\_R806\_70nt\_1mm\_db  
**-o** length forward read → 71  
**-e** length reverse read → 70  
**-t** customized taxonomic table → Silva\_taxonomic\_table  
**-q** ratio otu\_parent\_abundance/otu\_chimera\_abundance
(recommended 2, both OTU parents must be more than two times abundant
than the chimera → 2  
**-k** identity level between parents and chimera
(recommended 100, no error allowed, chimera as perfect combination of
two OTUs) → 100  
**-n** number of threads (for parallel computing) → 24

**Output:**

- A folder containing the reads included for every sample → total\_sample\_files
- A folder containing the OTUs before chimera checking for every sample → pre\_set\_otu\_files
- A folder containing the chimera uclust results for every sample → chimera\_uclust\_files
- A folder containing the OTUs for every sample → otus\_files
- A folder containing the OTUs for all the study → all\_otus\_file
- A folder containing the uclust results for all the OTUs against the 16S database → uclust\_results\_files
- A folder containing the complementary taxonomic assignment for all the OTUs → complementary\_tax\_files
- A folder containing the final taxonomic assignment for every sample → tax\_files

## 3.5. Make biom file

This script makes a biom file using a mapping file and a folder containing tax files.

```
make_biom_file.sh -m map_Mock_communities_V4.txt -t tax_files
```

**Input:**

**-m** mapping file address → map\_Mock\_communities\_V4.txt
**-t** folder containing the tax files → tax\_files

**Output:**

- A database containing every OTU present in any of the samples → map\_Mock\_communities\_V4\_otu\_database
- A fasta format database containing every OTU present in any of the samples → map\_Mock\_communities\_V4\_otu\_database.fa
- A database containing the taxonomic assignment of every OTU present in any of the samples → Map\_Mock\_communities\_V4\_tax\_database
- A biom file for the entire project → map\_Mock\_communities\_V4.biom

## 3.6. Create phylogenetic distance tree.

You can create the phylogenetic distance tree using clustalw (or any other multialignment tool).

The recommended parameters in clustalw are:

- Gap Opening Penalty: **3.00**
- Gap Extension Penalty: **1.00**
- Slow multiple alignment: **Yes**

**Input:**

OTU fasta file generate with make\_biom script → map\_Mock\_communities\_otu\_V4\_database.fa

**Output:**

- A file with the multiple alignment → map\_Mock\_communities\_V4\_otu\_database.aln
- A dnd tree file → map\_Mock\_communities\_V4\_otu\_database.dnd

# 3.7. Use QIIME for downstream analysis

Downstream analysis using QIIME could be performed with:

- map\_Mock\_communities.txt as mapping file.
- map\_Mock\_communities.biom as biom file.
- map\_Mock\_communities\_otu\_database.dnd as phylogenetic tree.

# 4. Optional scripts.

## 4.1. Compare different 16S rRNA gene regions.

This script allows for comparison of two regions by generating tax files with OTUs that have both,
the amplified region and the region you want to compare it with. The folders both\_regions\_tax\_files
generated for each of the regions should be merged and then this folder can be used for generating
the biom file.

**Only when two primer pairs are used.**

```
region_16S_comparator.sh -m Mock_communities_V4.txt -t tax_files -f primer_BSF784_76_nt_1mm_db -r primer_R1064_71_nt_1mm_db -p 1
```

```
region_16S_comparator.sh -m Mock_communities_V5V6.txt -t tax_files -f primer_F515_71nt_1mm_db -r primer_R806_70nt_1mm_db -p 2
```

**Input:**

**-m** mapping file address → Mock\_communities\_V4.txt or Mock\_communities\_V5V6.txt  
**-t** folder containing the tax files → tax\_files  
**-f** forward primer database address for the region you
want to predict (database adapted to the read length) →
primer\_F515\_71nt\_1mm\_db  
**-r** reverse primer database address for the region you
want to predict (database adapted to the read length) →
primer\_R806\_70nt\_1mm\_db  
**-p** position (1 to attach the predicted region after the OTU sequence and 2 to attach the predicted region before) → 1 or 2

**Output:**

- A folder with all the predicted sequences per sample → region\_prediction\_files
- A folder with tax files containing both regions (amplified sequence + predicted sequence) → both\_regions\_tax\_files

**A biom file can be created with both\_regions\_tax\_files**

```
make_biom_file.sh -m map_Mock_communities.txt -t both_regions_tax_files
```

## 4.2. Recover OTUs by pattern.

This script retrieves OTUs by pattern, this pattern can be searched in the sequence or in the
taxonomic assignment.

**Recover OTUs by sequence.**

```
otu_recovery_by_pattern.sh -t tax_files -n kmer -p GGCTGCG -s sequence
```

**Input:**

**-t** folder containing the tax files → tax\_files  
**-n** name of the searched pattern → kmer  
**-p** pattern to be retrieved → GGCTGCG  
**-s** search by taxonomy or sequence → sequence

**Output:**

- A folder otu\_retrievement\_files containing:
- A file with all the matching OTUs → kmer\_otus\_file
- A file with all the unique matching OTUs in fasta format → kmer\_unique\_otus\_file.fasta
- A file to be used for the alternative reassignment → kmer\_alternative\_taxonomy\_file

**Recover OTUs by taxonomy.**

```
otu_recovery_by_pattern.sh -t tax_files -n non_assigned -p NA -s taxonomy
```

**Input:**

**-t** folder containing the tax files → tax\_files  
**-n** name of the searched pattern → non\_assigned  
**-p** pattern to be retrieved (NA for non-assigned reads) → NA  
**-s** search by taxonomy or sequence → taxonomy

**Output:**

- A folder otu\_retrievement\_files containing:
- A file with all the matching OTUs → non\_assigned\_otus\_file
- A file with all the unique matching OTUs in fasta format → non\_assigned\_unique\_otus\_file.fasta
- A file to be used for the alternative reassignment → non\_assigned\_alternative\_taxonomy\_file

## 4.3. Assign new taxonomy with alternative classifier

The file with all the unique OTUs in fasta format can be used for taxonomical assignment using an
alternative classifier like BLAST or RDP classifier.  
This is useful to classify OTUs that could not be assigned using NG-Tax.  
This new taxonomy needs to be written in the 3 column of the alternative
reassignment file (non\_assigned\_alternative\_taxonomy\_file).  
OTUs can be also removed if the word “remove” is written in the 3rd column instead of a new taxonomy.

## 4.4. Reassignment or removal of OTUs

This script reassign OTUs to a new taxonomy introduced by the user in the 3 column of the
alternative reassignment file.  
If the word “remove” is introduced instead of new taxonomy the OTU will be removed.

```
taxonomical_reassignment.sh -a non_assigned_alternative_taxonomy_file -t tax_files
```

**Input:**

**-n** name used for the alternative reassignment file → non\_assigned\_alternative\_taxonomy\_file  
**-t** folder containing the tax files → tax\_files

**Output:**

- A folder containing the alternative taxonomic assignment for every sample → alternative\_reassigned\_tax\_files

**A biom file can be created with the alternative\_reassigned\_tax\_files folder.**

```
make_biom_file.sh -m map_Mock_communities.txt -t alternative_reassigned_tax_files
```

## 4.5. Assign taxonomy using NG-Tax

This script assigns taxonomy to a fasta file using NG-Tax.  
The name of the sequences should always starts by a number for example “>1\_sequence\_name”.

```
NG-Tax_taxonomic_classifier.sh -n theoretical_Mock_communities -f primer_F515_71nt_1mm_db -r primer_R806_70nt_1mm_db -o 71 -e 70 -t Silva_taxonomic_table
```

**Input:**

**-n** name of the file to be assigned [fasta] → theoretical\_Mock\_communities  
**-f** forward primer database address (database adapted to the read length) → primer\_F515\_71nt\_1mm\_db  
**-r** reverse primer database address (database adapted to the read length) → primer\_R806\_70nt\_1mm\_db  
**-o** length forward read → 71  
**-e** length reverse read → 70  
**-t** customized taxonomic table → Silva\_taxonomic\_table

**Output:**

- A folder containing the OTUs for every sample
- A folder containing the OTUs files → otus\_files\_theoretical\_Mock\_communities
- A folder containing the uclust results for all the OTUs against the
  16S database → uclust\_results\_files\_theoretical\_Mock\_communities
- A folder containing the complementary taxonomic assignment for all
  the otus → complementary\_tax\_files\_theoretical\_Mock\_communities
- A folder containing the final taxonomic assignment for every sample → tax\_files\_theoretical\_Mock\_communities

# 5. Citation.

See citation details at http://f1000research.com/articles/5-1791

The packages **QIIME** (if used for downstream analysis), **USEARCH** and **clustalw** should be also
cited.

Go

- © 2018 GitHub, Inc.
- Terms
- Privacy
- Security
- Status
- Help


- Contact GitHub
- Pricing
- API
- Training
- Blog
- About

You can’t perform that action at this time.

You signed in with another tab or window. Reload to refresh your session.
You signed out in another tab or window. Reload to refresh your session.


Press h to open a hovercard with more details.
