# Supplementary material for: NG-Tax, a highly accurate and validated pipeline for analysis of 16S rRNA amplicons from complex biomes
Source: F1000Res. 2018 Nov 23;5:1791. Originally published 2016 Jul 22. [Version 2] doi: 10.12688/f1000research.9227.2 (PMC6419982; doi:10.12688/f1000research.9227.2)
Supplement: Peer review history

# Peer review: Reviewer response for version 2

**DOI:** 10.5256/f1000research.18667.r43331

**Published:** 2019-03-13

**Authors:** Bonnie Hurwitz, George Watts

**Affiliations:** 1 The University of Arizona Cancer Center and Department of Pharmacology, The University of Arizona, Tucson, AZ, USA; 2 Department of Biosystems Engineering, The University of Arizona, Tucson, AZ, USA

The manuscript reports a new tool, NG-Tax, for analysis of 16S data which has been tested and benchmarked utilizing several mock communities. The manuscript is well written and clear. In particular, the introduction demonstrates the authors’ expertise and understanding of the issues and hurdles in analyzing 16S data. The data presented depicts the performance of NG-tax as compared to QIIME using default settings for both tools. As it stands, the manuscript is ready for indexing following the relatively minor comments below. There is one caveat, however, to our recommendation for indexing, stemming from the use of a now deprecated version of QIIME (version 1.X) for benchmarking. Since the initial submission of the manuscript in July 2016, a major revision of QIIME has been released (version 2.X) and therefore a more appropriate benchmark would be to compare against the latest version of QIIME. Importantly, some of the changes made in QIIME 2.X were to address the very problem that motivated the development of NG-Tax. A similar concern was raised by a previous reviewer (J. Tremblay), who criticized the use of QIIME with default settings given that these settings are known to be sub-optimal. The issues raised by default settings in QIIME have been examined by the QIIME team and optimal settings analyzed ^1^. Nonetheless, the authors’ response to Tremblay’s criticism applies to ours and therefore we don’t feel it is a requirement for recommending indexing. The real test of NG-tax will be when it is utilized by disparate researchers on real datasets over time, and thus dwelling on which is the most appropriate benchmark is beyond the scope of the current paper. Minor edits required/recommended before indexing are below:

 1. The authors state that RDP was replaced with SILVA SINA to classify sequences, however, the Figure 2 column heading reads SINA, while the Figure legend still lists RDP.

 2. Figure 4. The figure obfuscates the point by presenting too much material. Since the figure’s point is to show the poorer estimates of prevalence and mis-identifications in QIIME compared to NG-tax, it would be easier to see this point if there were fewer barcharts presented. Since any set of charts would suffice to make the point, we would recommend that the Reverse read barcharts (QIIME) be moved to supplemental data to simplify the figure. Similarly, move the V4 data to supplemental and only present V5-V6 for both NG-Tax and QIIME. Further, perhaps discuss an exemplary disparity between expected and observed in QIIME versus NG-Tax - especially one of mis-identification. Lastly, the figure legends do not provide enough information so that the figures stand alone without the manuscript text.  

 3. Figure 6. It is difficult to see that triangles are “darker”. We propose you omit the word “darker” in the legend and only call attention to the “circles” and “triangles” that distinguish the samples.

 4. Correct typo in the word “assess” in the following sentence in the introduction “still is no complete consensus about the best variable regions of the 16S rRNA gene to asses”. 

 5. In the “Barcoded PCR” methods, it is stated that 30 or 35 cycles of PCR were tested to quantify noise generated by the PCR protocol. The results are alluded to in the text as being presented in figure 6, however, it is unclear where they are shown as the figure only lists the four mock communities (MC1-4) and expected outcomes.

We have read this submission. We believe that we have an appropriate level of expertise to confirm that it is of an acceptable scientific standard.

## References

[1] BokulichNASubramanianSFaithJJGeversDGordonJIKnightRMillsDACaporasoJG: Quality-filtering vastly improves diversity estimates from Illumina amplicon sequencing. *Nat Methods*.2013;10(1) : 10.1038/nmeth.2276 57-9 10.1038/nmeth.2276 23202435PMC3531572

# Peer review: Reviewer response for version 2

**DOI:** 10.5256/f1000research.18667.r41304

**Published:** 2018-11-30

**Authors:** Fiona Fouhy

**Affiliations:** 1 Teagasc Food Research Centre, Fermoy, Ireland

I wish to approve the manuscript for acceptance as all of my previous comments have now been addressed.

I have read this submission. I believe that I have an appropriate level of expertise to confirm that it is of an acceptable scientific standard.

# Peer review: Reviewer response for version 1

**DOI:** 10.5256/f1000research.9931.r15389

**Published:** 2016-08-02

**Authors:** Fiona Fouhy

**Affiliations:** 1 Teagasc Food Research Centre, Fermoy, Ireland

This is a novel and important piece of research. Extensive research is being conducted using next generation sequencing but researchers are becoming increasingly aware that many factors such as PCR bias, region of the 16S rRNA gene targeted etc. can impact on the results achieved. This has a negative impact on the ability to compare results across studies. This manuscript sets about to address this with their new analysis pipeline NG-Tax.

 The title of the manuscript is good.

 The abstract accurately summarises the research but the results section should have less methods and more results.

 Figure 1 is vague and fails to show the unique aspects of how NG-Tax differs from e.g. QIIME. More details would make this figure useful.

 I think greater details on the filtering and the classification used by this approach would benefit the reader. Perhaps a table showing the differences between this approach and e.g. RDP , QIIME etc. would improve the readers ability to interpret the novelty of the work.

 This work was done only using HiSeq data. Do the authors feel that the approach would be equally successful on approaches e.g. Ion, MiSeq etc where longer reads are achieved? It would also be nice to test the approach with a real life data set and not a mock community and see how the results compare to those achieved using traditional analysis approaches.

 Figures 3 and 4 are difficult to interpret, perhaps remake as tables.

I have read this submission. I believe that I have an appropriate level of expertise to confirm that it is of an acceptable scientific standard, however I have significant reservations, as outlined above.

# Response

**Published:** 2018-11-14

**Authors:** Javier Ramiro-Garcia

**Affiliations:** University of Luxembourg, Luxembourg

**This is a novel and important piece of research. Extensive research is being conducted using next generation sequencing but researchers are becoming increasingly aware that many factors such as PCR bias, region of the 16S rRNA gene targeted etc. can impact on the results achieved. This has a negative impact on the ability to compare results across studies. This manuscript sets about to address this with their new analysis pipeline NG-Tax.**

**The title of the manuscript is good.**

Thank you.

**The abstract accurately summarizes the research but the results section should have less methods and more results.**

We tried to find a proper balance between results and methods but taking into account that the paper describes a tool, a description of it should be included because the pipeline is both method and results at the same time. But we tried to include those results needed to prove that NG-Tax is suitable for 16S amplicon analysis:

1) Taxonomy assignment using short reads should be comparable with the assignment using the complete 16S rRNA gene.

2) Composition profiles based on sequencing data should resemble the real composition of the biological sample.

3) α and β diversity should match the expected α and β diversity.

4) Results should be reproducible and therefore robust against biological variation (different sample compositions) and technical (PCR and sequencing settings) biases.

We consider that these requirements were met by NG-Tax and hope that they will convince readers of the actual improvements that were made, regarding robustness against methodological aspects as well as a more accurate reproduction of the MC compositions. In the new version of the manuscript we included more statistical tests to measure accuracy and reproducibility of NG-Tax.

**Figure 1 is vague and fails to show the unique aspects of how NG-Tax differs from e.g. QIIME. More details would make this figure useful.**

As suggested by the reviewer Figure 1 now includes those unique aspects of NG-Tax.

**I think greater details on the filtering and the classification used by this approach would benefit the reader. Perhaps a table showing the differences between this approach and e.g. RDP , QIIME etc. would improve the readers ability to interpret the novelty of the work.**

As suggested by the reviewer we detailed the filtering and the classification process in the user manual for those readers that want to dive into the technical details.

“This script demultiplexes the raw data into samples using the information contained in the mapping file. It also generates an OTU table per sample after removing chimeras and assigns taxonomy to the OTUs. NG-Tax is designed for short reads, 70 nucleotides is the recommended read length. Reads can be trimmed to this length by the script. Longer length can be selected by the user but comparison with 70 nucleotide analysis is advisable.

OTU picking: For each sample reads are ranked by abundance and OTUs are added to an OTU table starting from the most abundant sequence until the read abundance is lower than a percentage defined by the user (recommendeded is at is 0.1%). Subsequently, the discarded reads are clustered to the OTU table allowing one mismatch.

Chimera removal: OTUs are subjected to non-reference based chimera checking according to the following principle: given three OTUs named A, B and C, C will be considered a chimera when the following conditions are satisfied: C and A 5’ reads are identical, C and B 3’ reads are identical and both OTUs, A and B, are at least twice as abundant as OTU C.

Taxonomic assignment: For each OTU, usearch is used to retrieve hits for the forward and reverse reads against their respective trimmed reference database.

Hits that are in common between both reads are divided in 6 identity thresholds 100, 98, 97, 95, 92, 90.

A hit belongs to a certain level, for example 97, when both reads have at least a 97 percentage identity with that hit.

Using the highest available identity threshold, NG-Tax assigns the consensus taxonomy to the OTU if this taxonomy is supported for at least half of the hits.

Genus, Family or Order remains unassigned if the maximum identity percentage level is lower or equal to 97%, 95% and 92% respectively.”

Now we also included the main characteristics of NG-Tax in Figure 1.

The OTU generation is the main difference between NG-Tax and QIIME. NG-Tax uses a *de novo *generation approach without clustering. This increases the resolution and allows for the distinction between OTUs with one nucleotide distance. In addition, NG-Tax generates OTUs independently for each sample, which avoids problems associated to sample size heterogeneity. Another important feature of NG-Tax is the use of non-fixed thresholds for the taxonomic assignment, which results in more accurate classifications. To highlight those points we added the text “No clustering → max resolution” to Figure 1 and indicated in the workflow that OTUs are generated per sample. We also clearly state that NG-Tax does not use any clustering and explain that the taxonomic classification uses different identity levels.

The authors of the RDP classifier stated that it does not perform well for short sequences, i.e. a length of 200 nt would give accurate family level classification but shorter reads will not, likely due to insufficient features ^1^. In contrast, NG-Tax has been specifically designed for short reads.

**This work was done only using HiSeq data. Do the authors feel that the approach would be equally successful on approaches e.g. Ion, MiSeq etc where longer reads are achieved? It would also be nice to test the approach with a real life data set and not a mock community and see how the results compare to those achieved using traditional analysis approaches.**

NG-Tax has been the reference method for 16S rRNA gene amplicon analysis in our lab for more than two years now, and has been used in more than 30 manuscripts that have been submitted or are in preparation. One of these manuscripts ^1^ was published before this manuscript. Since then another fifteen studies using NG-Tax have been published ^2-16^. These studies contain biological samples that belong to very different and specific environments and were sequenced both on MiSeq and HiSeq instruments. These will contribute to the assessment of NG-Tax’s performance, however these were not included in the current manuscript since the data is accessible in the aforementioned publications.

**Figures 3 and 4 are difficult to interpret, perhaps remake as tables.**

With Figure 3 and 4 we intended to visualize how close the observed sample composition resembled the expected composition at a glance and how many different taxa are found in the data. In order to further improve interpretation, we have now also added Table 1 to provide detailed information as to the number of misclassified reads at different taxonomic levels and Figure 5, which shows boxplots of the distances to the expected composition. We also performed statistical tests to quantitatively compare the performance of NG-Tax and QIIME. As suggested by the reviewer we included the tables with the taxonomical profiles as supplementary data, which can be used for evaluation of the results.

# Peer review: Reviewer response for version 1

**DOI:** 10.5256/f1000research.9931.r15175

**Published:** 2016-08-02

**Authors:** Julien Tremblay

**Affiliations:** 1 Biomonitoring, National Research Council Canada, Montreal, QC, Canada

This paper describes a pipeline for processing 16S rRNA amplicon data. They implemented an experimental design in which they used data coming from three different HiSeq2000 runs using two variable regions (V4 and V5-V6). It is however not clear if their data has been generated in-house or if their data was actually coming from public databases. This should be explicitly stated somewhere (unless I missed it). Using this data as input, the authors developed a pipeline labeled NG-Tax, which according to them: 1) better accounts (compared to what?) for errors associated with a range of technical aspects of 16S rRNA amplicon sequencing and 2) improves comparability be removing technical bias and facilitating efforts towards standardization.  In my view, the problem is that why their pipeline does 1) and 2) is not addressed in depth. The description of the technical aspects of their pipeline in the first part of the result section only very summarily describes the general workflow of the pipeline, but nowhere do they describe how exactly OTU picking is done (see comment below). How exactly Chimera are detected? With an open-source package? In-house script? Taxonomic assignment methodology is unclear as well. The authors state that they are using uclust for taxonomic assignment, while uclust is a sequence clustering software (also see comments below).

 Then the authors compares their pipeline results with the ones generate by Qiime with default paramters. Qiime with its default parameters is already known to not perform optimally (See UPARSE paper, Edgar, 2013). I think that comparing with Qiime for validation is okay, but do not spend too much time dissecting the results. What the authors should focus on is, I think, on improving substantially on the technical description of their pipeline – describe each step in details. If open source packages are being used, say so, if not, describe your script/software/algorithm. Also please make the source code available under a code repository (Github or Bitbucket for instance).

 In my view the paper is not acceptable in its current form.

 Specific comments:

- At the sentence "mostly because available 16S rRNA gene reference databases were thought to provide insufficient coverage13–16." Can you please elaborate on that? What do exactly mean by that?
- "there still is no standard or consensus of best choices for variable regions."
  I don't fully agree with this. Depending on your field of study, a certain consensus can usually be found. For instance, the Earth Microbiome project recommends two primer sets (V4 and the 'newer' V4-V5) - Many labs investigating soil or environmental samples in general will effectively favor these primers because they are being used by a large part of the community which readily enables inter-lab community/study comparisons.
- Concerning the OTU picking section: It is not clear how exactly you pick your OTUs. Basically, you are kind of dereplicating/clustering your raw reads data set at 100% ID and then create a one column OTU table for each sample? Please clarify.
- “Phred score, such as minimum average Phred score, maximum number of ambiguous positions, maximum bad run length, trimming and minimum read length after quality trimming, are not utilized in NG-Tax because quality scores from the Illumina base caller have been shown to be of limited use for the identification of actual sequence errors for 16S rRNA gene amplicon studies9,37.”
  Yes Q scores have their limitation, but it is unwise to not filter for reads containing Ns and reads of very poor Q scores. Some basic filtering should be implemented to at least filter for very bad data. For instance if you have a read with 10 bases with Q score lower than 10, this read should obviously be removed.
- “To speed up the procedure by several orders of magnitude, 16S rRNA gene sequences from the reference database are trimmed to contain only the region amplified by the primers.”
  Please specify how you generated your trimmed database of 16S rRNA genes ref. In silico PCR? A multiple alignment that was trimmed at specific coordinates?
- "In the current version of NG-Tax, taxonomy is assigned to OTUs utilizing the uclust algorithm16 and the Silva_111_SSU Ref database, containing 731,863 unique full length 16S rRNA gene sequences. To ensure maximum resolution and avoid the risk of errors due to clustering-associated flaws ( *e.g. *reference sequence error hotspots, overrepresentation of certain species and lack of robustness in cluster formation by clustering algorithms),we use the non-clustered database. To speed up the procedure by several orders of magnitude",
  Uclust is for clustering sequences/reads and not for taxonomic assignment…? Taxonomic assignment is done by other means (RDP classifier), but certainly not with uclust.
- For each OTU, a taxonomic assignment is retrieved at six different identity thresholds levels (100%, 98%, 97%, 95%, 92% and 90%) and at two taxonomic levels (genus and family).
  How exactly are OTUs classified? With an in-house method? The RDP classifier? Please elaborate.
- Figure 1. Please add more details. Are you using open-source packages in your pipeline? If so please indicate.
- Table 1: Table 1 is heavy and not really meaningful. Would fit in more appropriately in suppl. material.
- Figure 3 and 4: Please find another way of displaying data of figure 3. It is simply not feasible to associate a color to a given bar graph. Maybe consider using a heatmap with hierarchical clustering or a PCA/PCoA? Typically for taxonomiy stacked barplots you can’t really go above 20 different colors. After that it becomes indistinguishable.
- "Because the focus of NG-Tax is to retain as much biological signal as possible while minimizing the impact of any technical choice,"
  But how exactly does NG-Tax retain more biological signal than other pipelines, what does that mean?
- Discussion: The authors say that their pipeline outperforms Qiime, but nowhere is discussed how exactly does Qiime works. How exactly does Qiime generate OTUs, how are the reads QCed? How is the classification performed, what training sets are being used for classification? It is already known that Qiime does not perform well with default parameters (see R. Edgar’s UPARSE paper), so Qiime does not represent a gold standard, especially with default parameters.
- NG-Tax pipeline availability. Please include the pipeline on a Github or bitbucket repository.

I have read this submission. I believe that I have an appropriate level of expertise to state that I do not consider it to be of an acceptable scientific standard, for reasons outlined above.

## References

[1] EdgarRC: UPARSE: highly accurate OTU sequences from microbial amplicon reads. *Nat Methods*.2013;10(10) : 10.1038/nmeth.2604 996-8 10.1038/nmeth.2604 23955772

# Response

**Published:** 2018-11-14

**Authors:** Javier Ramiro-Garcia

**Affiliations:** University of Luxembourg, Luxembourg

**This paper describes a pipeline for processing 16S rRNA amplicon data. They implemented an experimental design in which they used data coming from three different HiSeq2000 runs using two variable regions (V4 and V5-V6). It is however not clear if their data has been generated in-house or if their data was actually coming from public databases. This should be explicitly stated somewhere (unless I missed it).**

To further clarify this we added the section Datasets:

Datasets:

Four synthetic communities of varying complexity were created, consisting of 16S rRNA gene amplicons of phylotypes (PTs) associated with the human GI-tract (Dataset 1). This specific setup limited the likelihood of overfitting to a particular OTU composition or distribution and allowed us to assess (1) the quantification potential, (2) noise floor and (3) the effect of richness and diversity on quality filtering parameters, thus ensuring a higher fidelity with biological samples than by using a single MC. As a reference, to assess the quality of the taxonomic classifications, full length sequences for all PTs were obtained through Sanger sequencing. Expected MCs were created by trimming the full length sequences to the sequenced region. MC1 and MC2 consisted of equimolar amounts of 17 and 55 PTs, respectively. MC3 contained 55 PTs in staggered concentrations typical for the human GI-tract, and MC4 included 50 PTs with relative abundances ranging between 0.001 and 2.49%. To account for pipetting errors, each of the four MCs was produced in triplicate. To design a pipeline that puts more focus on biology, these 12 MC templates were used to sequence the MCs with different conditions that cover most of the technical bias associated with 16S rRNA gene amplicon studies reported in literature. To this end, we:

1. Targeted either region V4 or region V5-V6,
2. Used four PCR protocols differing in the number of PCR cycles and reaction volumes.
3. PCR products were analysed in three different sequencing runs and in seven different libraries.
4. Two different library preparation protocols (with and without an additional amplification of 8 cycles) were applied (Dataset 1).

In addition the sequencing depth ranged from 2363 to 335822 reads per sample (Dataset 1). One phylotype, PT17 ( *Parabacteroides*), attracted so much sequencing error in the V4 region that it was rendered undetectable although it was amplified by the primers (Supplementary Figure 1). Therefore, to test both pipelines without this sequencing anomaly, it was removed from the analysis.

In this section we explain how we created and sequenced the MCs. The sequencing data was generated by a sequencing company (GATC, Constance, Germany; see section Materials and Methods). The sequencing data has been submitted to the ENA repository, and we added the following sequence data availability section:

Sequence data availability:

Sequence data have been deposited in the European Nucleotide Archive ^46^, accession number [ENA:PRJEB11702]) http://www.ebi.ac.uk/ena/data/view/PRJEB11702 (amplicon sequencing data for all 49 samples) and [ENA:LN907729-LN907783]) (full length 16S rRNA gene sequences for all 55 Pts).”

**Using this data as input, the authors developed a pipeline labeled NG-Tax, which according to them: 1) better accounts (compared to what?) for errors associated with a range of technical aspects of 16S rRNA amplicon sequencing and 2) improves comparability be removing technical bias and facilitating efforts towards standardization. In my view, the problem is that why their pipeline does 1) and 2) is not addressed in depth.**

We agree with the reviewer that the highlighted elements were not sufficiently clear and lacked an explanation why we believe that NG-Tax performs better. Therefore we replaced this sentence with:

”This allowed for the development of NG-Tax, a pipeline that accounts for biases associated with this range of technical aspects associated with 16S rRNA gene amplicon sequencing. Therefore NG-Tax will improve comparability by removing technical bias and facilitate efforts towards standardization, by focusing on reproducibility as well as accuracy. To assess the performance regarding key output parameters such as taxonomic classification, composition and richness, and α and β diversity measures, we benchmarked the results obtained with NG-Tax.”

In order to account for errors and increase comparability by removing technical bias from 16S rRNA amplicon studies, NG-Tax should fulfill the following requirements:

1. Taxonomy assignment using short reads should be comparable with the assignment using the complete 16S rRNA gene.
2. Composition profiles based on sequencing data should resemble the real composition of the biological sample.
3. α and β diversity should match the expected α and β diversity.
4. Results should be reproducible and therefore robust against biological variation (different sample compositions) and technical (PCR and sequencing settings) biases.

We consider that these requirements were met by NG-Tax, as supported by the following data.

Figure 2 shows the high similarity of the taxonomic classification of the V4 and V5V6 amplicon results compared to full length sequences using SILVA Incremental Aligner (SINA). The specificity and the number of hits testify to the reliability of the assignments.

Table 1 shows the low number and percentage of spurious reads.

Figure 3 shows that NG-Tax derived compositional profiles based on sequencing data accurately resemble the expected profiles.

Figure 5 quantifies the distances to the expected profiles.

Figure 6 & 7: the PCoA plots show that MCs group by type, despite all technical bias associated with 16S rRNA gene amplicons sequencing, such as PCR settings, and primer or region selection. Figure 7 shows that all within-MC pairwise comparisons and the dispersion of all pairwise comparisons are significantly smaller in NG-Tax meaning that distances within and between MC types are robust. These results could not have been achieved without a proper reduction of the aforementioned biases. This will improve comparability by enabling direct comparison between studies even when using slightly different approaches.

**The description of the technical aspects of their pipeline in the first part of the result section only very summarily describes the general workflow of the pipeline, but nowhere do they describe how exactly OTU picking is done (see comment below). How exactly Chimera are detected? With an open-source package? In-house script? Taxonomic assignment methodology is unclear as well. The authors state that they are using uclust for taxonomic assignment, while uclust is a sequence clustering software (also see comments below).**

In the revised manuscript we substantially increased the amount and detail of information on the description of the general work flow. All the critical steps, including barcode & primer filtering, OTU picking, mapping rejected reads to accepted OTUs, *de novo* chimera filtering, taxonomic assignment and the generation of a phylogenic tree are now detailed in Figure 1 and explained in the user manual.

**Then the authors compares their pipeline results with the ones generate by Qiime with default paramters. Qiime with its default parameters is already known to not perform optimally (See UPARSE paper, Edgar, 2013). I think that comparing with Qiime for validation is okay, but do not spend too much time dissecting the results.**

We agree with the reviewer’s view on the default parameters of QIIME, however, the major improvements are gained by not clustering and processing the reads per sample. Therefore, the presented results cannot be achieved with QIIME independent of the parameters we choose. Besides that, testing QIIME under different settings has been already extensively covered elsewhere ^5^ and if we would change parameters, reviewers could argue that our chosen parameters are less than optimal and therefore we stayed with the default settings.

Nonetheless, as suggested by the reviewer we reproduced the QIIME analysis with a 0.1% abundance threshold, and this is now included in the supplementary data. The results using 0.1% or 0.005% are consistent and show no performance gain (“Supplementary data. QIIME beta-div results all settings”). This is also in line with the result shown by Bokulich et al 2013 ^1^, supplementary material 2; pages 8, 9 and 10. This text includes a comparison of the expected composition against real sample composition using different filtering parameters. One of these parameters is OTU abundance and the plot shows that the obtained profiles do not change much using different filtering abundance thresholds.

Although we agree with the reviewer that we need not to put too much emphasis on the QIIME results, they do show the consequences when technical bias is not adequately taken care of, which makes it easier for the non-technical reader to place the results achieved by NG-Tax into context.

**What the authors should focus on is, I think, on improving substantially on the technical description of their pipeline – describe each step in details. If open source packages are being used, say so, if not, describe your script/software/algorithm. Also please make the source code available under a code repository (Github or Bitbucket for instance).**

**In my view the paper is not acceptable in its current form.**

We thanks the reviewer for the constructive suggestions and hope that the changes introduced in the manuscript and supplementary data help to change his opinion.

As suggested by the reviewer the source code is now available on Github ( https://github.com/JavierRamiroGarcia/NG-Tax.git).

**Specific comments:**

**At the sentence "mostly because available 16S rRNA gene reference databases were thought to provide insufficient coverage 13–16." Can you please elaborate on that? What do exactly mean by that?**

In the past, when pyrosequencing was the standard sequencing method, open reference base OTU picking was the common strategy for two main reasons: 1) reference databases were still very small and thought to provide insufficient coverage and 2) the more computationally intense open reference methods were used because the amount of reads generated were lower than nowadays. Over time databases were more complete and the amount of data generated increased the needed computational time, so close reference OTU picking gained popularity. Currently, with new bioinformatics solutions, open reference OTU picking is gaining ground and NG-Tax is following that trend by implementing a new open reference OTU picking algorithm.

**"there still is no standard or consensus of best choices for variable regions."**

**I don't fully agree with this. Depending on your field of study, a certain consensus can usually be found. For instance, the Earth Microbiome project recommends two primer sets (V4 and the 'newer' V4-V5) - Many labs investigating soil or environmental samples in general will effectively favor these primers because they are being used by a large part of the community which readily enables inter-lab community/study comparisons.**

We agree with the reviewer that there is a certain consensus for some projects, but still there are many publications addressing the differences in results when different primers are used. Therefore, when choosing a primer pair, whether these primers are used by the community becomes an important factor if afterwards the researcher wants to compare the results with existing studies. The idea of NG-Tax is to decrease the importance of this factor by providing comparable results across different primer sets, giving more freedom to the researcher to explore new possibilities. But as suggested by the reviewer we softened our statement and rephrased as:

“There still is no complete consensus regarding best choices for variable regions even if some initiatives like the Earth Microbiome Project are setting standards that are increasingly being adopted by the field.”

**Concerning the OTU picking section: It is not clear how exactly you pick your OTUs. Basically, you are kind of dereplicating/clustering your raw reads data set at 100% ID and then create a one column OTU table for each sample? Please clarify.**

We tried to make the paper as readable as possible by not adding too much technical information. Realizing that we have excessively reduced technical detail in the original manuscript, in the new version all technical details can be found in the user manual. An indication that this information can be found in the user manual is now included in the manuscript.

In NG-Tax OTUs are generated per sample using the following strategy:

For each sample reads are ranked by abundance and OTUs are added to an OTU table starting from the most abundant sequence until the read abundance is lower than a percentage defined by the user (recommended is at is 0.1%). Subsequently, the discarded reads are clustered to the OTU table allowing one mismatch.

In practical terms this is in fact guided clustering where seeds are determined by abundance. The difference with an normal clustering approach is that there is no clustering to define the seeds. This allows seeds that differ as little as one nucleotide. The clustering is applied only afterwards to compensate for potential bias due to PCR and sequencing errors. Error is sequence-specific, and hence some sequences could be affected more than others. If a species specific amplicon is more prone to PCR or sequencing errors, the relative abundance of that particular OTU will be underestimated. But after clustering, OTUs more prone to error receive a higher percentage of discarded reads than others, this differential recovery helps to reestablish the true composition that was lost due to sequence specific error rates.

**“Phred score, such as minimum average Phred score, maximum number of ambiguous positions, maximum bad run length, trimming and minimum read length after quality trimming, are not utilized in NG-Tax because quality scores from the Illumina base caller have been shown to be of limited use for the identification of actual sequence errors for 16S rRNA gene amplicon studies9,37.”**

**Yes Q scores have their limitation, but it is unwise to not filter for reads containing Ns and reads of very poor Q scores. Some basic filtering should be implemented to at least filter for very bad data. For instance if you have a read with 10 bases with Q score lower than 10, this read should obviously be removed.**

We fully agree with the reviewer. A filtering process is needed, and this is already implemented in NG-Tax. The point is that it is not based in quality score but based on abundance. Illumina have reported that 95%-97% of the reads have Q>30 ( http://www.illumina.com/documents/products/technotes/technote_Q-Scores.pdf). This 3 to 5 percent of reads with lower quality will contain reads for all the different phylotypes, and within phylotypes there will be reads with errors in different positions and with different base substitutions. This decreases the probability of having exactly the same erroneous read. Therefore we expect that any specific erroneous read should be in low abundance. Subsequently, when samples are filtered by discarding low abundance sequences, those low quality reads will be removed without the need to check for quality scores.

In addition quality scores do not account for PCR errors since the base caller will give them very high scores, because according to the sequencer they are real sequences. In contrast, filtering by abundance is insensitive to the error source, and hence if the reads with PCR errors are in low abundance (especially if high fidelity taq polymerase is used), they will be also removed.

A good example of how stringent quality thresholds can bias the results can be found in Bokulich et al 2013, supplementary material 2; pages 8, 9 and 10 ^1.^

**“To speed up the procedure by several orders of magnitude, 16S rRNA gene sequences from the reference database are trimmed to contain only the region amplified by the primers.”**

**Please specify how you generated your trimmed database of 16S rRNA genes ref. In silico PCR? A multiple alignment that was trimmed at specific coordinates?**

**We thank the reviewer for the suggestion. This information is important and now it has been added to the user manual. NG-Tax applies an in silico PCR using the primers and a reference database given by the user. Degenerated primer positions are allowed and alternative primers with mismatches can be supplied.**

**"In the current version of NG-Tax, taxonomy is assigned to OTUs utilizing the uclust algorithm16 and the Silva_111_SSU Ref database, containing 731,863 unique full length 16S rRNA gene sequences. To ensure maximum resolution and avoid the risk of errors due to clustering-associated flaws (e.g. reference sequence error hotspots, overrepresentation of certain species and lack of robustness in cluster formation by clustering algorithms),we use the non-clustered database. To speed up the procedure by several orders of magnitude",**

**Uclust is for clustering sequences/reads and not for taxonomic assignment…? Taxonomic assignment is done by other means (RDP classifier), but certainly not with uclust.**

**For each OTU, a taxonomic assignment is retrieved at six different identity thresholds levels (100%, 98%, 97%, 95%, 92% and 90%) and at two taxonomic levels (genus and family).**

**How exactly are OTUs classified? With an in-house method? The RDP classifier? Please elaborate.**

First we wanted to inform that uclust has been substituted by usearch in the scripts for the second version of the manuscript.

Any or at least most methods for taxonomic assignment contain two main steps. First, the read to be classified is linked to sequences in a reference database by sequence similarity, and then the taxonomic information of linked sequences, termed hits, is transferred to the sequence to be classified. Different methods can be used to perform the linking step. In our case we used usearch (previously uclust). We used dynamic thresholds to get hits at 6 different identity levels, after which the taxonomic information is transferred to the read of unknown taxonomy by the NG-Tax classifier algorithm. Similar dynamic thresholds are used by rtax ^2^.

A description of how the NG-Tax classifier works:

For each OTU, usearch is used to retrieve hits for the forward and reverse reads against their respective trimmed reference database. Hits that are in common between both reads are divided in 6 identity thresholds 100, 98, 97, 95, 92, 90. A hit belongs to a certain level, for example 97, when both reads have at least a 97 percentage identity with that hit. Using the highest available identity threshold, NG-Tax assigns the consensus taxonomy to the OTU if this taxonomy is supported for at least half of the hits. Genus, Family or Order remains unassigned if the maximum identity percentage level is lower or equal to 97%, 95% and 92% respectively. The levels lower than 97% are only useful for unexplored environments; otherwise most of the OTUs are assigned at 100% identity.

As suggested by the reviewer we have included a detailed explanation of how the algorithm works in the user manual provided in the supplementary files.

**Figure 1. Please add more details. Are you using open-source packages in your pipeline? If so please indicate.**

We thank the reviewer for the suggestion, now we added a figure with more details. As stated in the user manual we use USEARCH and QIIME.

**Table 1: Table 1 is heavy and not really meaningful. Would fit in more appropriately in suppl. material.**

As suggested by the reviewer the table 1 is now supplied as supplementary material.

**Figure 3 and 4: Please find another way of displaying data of Figure 3. It is simply not feasible to associate a color to a given bar graph. Maybe consider using a heatmap with hierarchical clustering or a PCA/PCoA? Typically for taxonomy stacked barplots you can’t really go above 20 different colors. After that it becomes indistinguishable.**

With figure 3 and 4 we just wanted to show in one glance, how close the sample compositions resembled the expected composition and how many different taxa are found in the data. In the new version of the manuscript we have added boxplots showing distances to the expected profiles to improve interpretation. An excel file with taxonomic profiles is also added to the supplementary material for further interpretation.

PCoA plots showing distances between samples and expected for both pipelines are provided in figure 6. Figure 7 shows those distances as pairwise comparisons.

**"Because the focus of NG-Tax is to retain as much biological signal as possible while minimizing the impact of any technical choice,"**

**But how exactly does NG-Tax retain more biological signal than other pipelines, what does that mean?**

We agree with the reviewer that the sentence is confusing. Therefore it has been rephrased.

”Therefore, these indices probably provide a better estimate of the true diversity for data generated by high throughput next generation technology sequencers.

Because the aim of NG-Tax is to enhance the biological signal as much as possible by minimizing the impact of any technical choice, divergence-based α-diversity (Phylogenetic Diversity (PD) [41]) and β-diversity (Unifrac [39]) metrics were used to visualize the diversity within and between MCs (Figure 6)”.

**Discussion: The authors say that their pipeline outperforms Qiime, but nowhere is discussed how exactly does Qiime works. How exactly does Qiime generate OTUs, how are the reads QCed? How is the classification performed, what training sets are being used for classification? It is already known that Qiime does not perform well with default parameters (see R. Edgar’s UPARSE paper), so Qiime does not represent a gold standard, especially with default parameters.**

In our manuscript we applied recommended settings like those described in the Bokulich paper. This paper extensively describes QIIME, the rationale behind the recommendations and the way that these choices impact the data. The scope of this manuscript was not to test QIIME under different settings. For NG-Tax analysis we also employed default and recommended settings so we thought that even if it is not optimal and has limitations, this could be a fair approach.

We also analyzed the MCs with QIIME to show that this dataset is not an exceptional case with regards to the commonly reported problems (such as many un- or poorly classified OTUs, inflated richness and diversity, taxonomic profiles that do not match the expected ones, region dependent taxonomic classification and results which are highly dependent on minor changes in the experimental procedures) are also found in this dataset. So in our mind the QIIME analysis should primarily be seen as a performance comparison. In fact we encourage researchers to use more than one method, as this will increase the amount of information they can obtain from their datasets and determine the quality of their data. This will benefit their research and by extension the whole field.

Nevertheless in an effort to increase comparability we also performed an additional analysis using QIIME with a 0.1% abundance threshold (which is conservative compared to the advised setting of 0.005%). Nevertheless this did still not reproduce the biological signal and the results obtained with 0.1% or 0.005% are consistent. These analyses have been added to the supplementary material as “Supplementary data. QIIME beta-div results all settings”.

**NG-Tax pipeline availability. Please include the pipeline on a Github or bitbucket repository.**

NG-Tax scripts were previously available as supplementary material, and as suggested by the reviewer they are now also available in Github ( https://github.com/JavierRamiroGarcia/NG-Tax.git)

References

1. Bokulich NA, Subramanian S, Faith JJ, et al. Quality-filtering vastly improves diversity estimates from Illumina amplicon sequencing. Nat Methods 2013;10:57-9.

2. Soergel DA, Dey N, Knight R, Brenner SE. Selection of primers for optimal taxonomic classification of environmental 16S rRNA gene sequences. ISME J 2012.

# Peer review: Reviewer response for version 1

**DOI:** 10.5256/f1000research.9931.r15177

**Published:** 2016-08-02

**Authors:** Thomas S. B. Schmidt

**Affiliations:** 1 Institute of Molecular Life Sciences, University of Zurich, Zürich, Switzerland

In their manuscript, the authors introduce NG-Tax, an open-source software for the (meta-)analysis of 16S rRNA-based microbiome datasets. Their tool focuses on an important and so-far arguably understudied aspect of microbial ecology research: the integration of results across studies, in view of both technical and biological variation.

 The approach is interesting and addresses important points. In particular, several sequencing datasets of different mock communities were generated, even using different primer sets: this is great data to benchmark on, and many (most) other papers introducing tools do not provide benchmarks on such an array of real (mock) data. In general, I feel that this is very interesting work and that NG-Tax can be a promising alternative to existing tools in the field.

 However, there are several points that I feel would need to be addressed in order for the manuscript to stand tall, and for the reader to get a good understanding of how NG-Tax can be useful in practice.

 Major comments:

- Even after reading the manuscript and online user manual repeatedly, I have to admit that it is not completely clear to me how NG-Tax works in detail, and in which points exactly it differs from existing approaches. Based on the introduction, I gather that NG-Tax relies on closed-reference OTU picking, but this is not mentioned explicitly anywhere in the text. Also, does reference-based OTU picking in NG-Tax rely on uclust? If yes, which version and parameters were used, and how do they differ from QIIME’s defaults? Also, the Background and Discussion sections do not elaborate on the various disadvantages of closed-reference approaches; most importantly, closed-ref only takes into account sequences matching the database and removes everything else. When integrating sequence data from different primer sets, this is arguably the most straightforward approach; however, the limitations should be discussed.
- I gather from the text that NG-Tax’s main innovations are the use of primer-tailored reference databases and a different (more conservative) read abundance filtering scheme. It is perfectly valid to benchmark these against QIIME’s default settings; however, it would be great to see how QIIME performs with similarly conservative settings, to better understand where NG-Tax’s edge in performance comes from.
- Regarding taxonomy assignments, it is valid to compare NG-Tax’s uclust-based approach to QIIME’s uclust-based approach. However, I believe that the gold standard continues to be the RDP Classifier, and it would be interesting to see a performance comparison to this tool (on the short-read data, not only on full-length reads). Also, how does taxonomic classification by NG-Tax differ conceptually from RTAX ( http://www.uio.no/english/services/it/research/hpc/abel/help/software/rtax.html)? I do believe that they are not equivalent, but the approaches appear somewhat related.
- In general, the results on taxonomic classification are not discussed quantitatively. From Figures 3&4, the visual impression is that NG-Tax indeed better approximates expected taxonomic profiles than QIIME, but it is hard to quantify this from stacked bar charts. I would suggest to compute e.g. Euclidean or more sophisticated distances of classified taxonomic profiles to the expected distribution. Also, it would be interesting to see quantitative sensitivities and specificities (or F1-scores?) on the taxonomic assignments; particularly also when running on the exact same (more conservatively filtered) dataset for QIIME. Some numbers on specificity are provided in the Abstract and Conclusion sections – but I am not sure if specificity may be gained at the expense of sensitivity based on the more rigid read filtering upstream.
- As a suggestion, but certainly not as a request, I would recommend to maybe include additional, independent datasets to benchmark on. For example, Tremblay *et al. *(2015) have published data on mock communities sequenced with different primer sets and on different platforms. Such data could contribute to a yet more general assessment of NG-Tax performance.

Minor comments (chronologically, not in order of importance): 

- Background, “The consequence of this approach is that the ‘quality’ of the clustering of the reference set propagates to reference-picked OTUs.” I believe that as such, this statement is not fully valid or supported. In fact, the negative complement is arguably true: reference-based OTU picking against a “bad” reference can never provide “good” OTUs (a garbage-in, garbage-out problem, so to say). However, even with a good reference, a bad mapping algorithm can generate non-informative reference-based OTU sets. Schloss & Westcott have recently published a study which discusses this point, among others (Westcott & Schloss, 2015).
- Background, “However, in 16S rRNA gene amplicon sequencing every sequencing error could potentially lead to the false discovery of a new species.” I have two comments on this statement. First, I believe that the term “species” in this context can be misleading and I feel that the neutral term OTU or diversity unit would be more appropriate. Second, there is a large body of literature on how sequencing errors affect 16S-based diversity studies beyond the cited Bokulich et al paper (starting from Kunin *et al.*, 2010), and it would be worth to at least mention these, although an in-depth discussion would probably lead away from this study’s focus. Also, it may be worth mentioning recent algorithmic approaches to tackling this issue, such as DADA2 (Callahan *et al*., 2016).
- Results & Discussion, chimera filtering. The implemented method for chimera filtering appears a little *ad hoc *and heuristic, although the proposed approach certainly makes sense intuitively. However, given the long history of “chimera-slaying” algorithms and the quite sobering benchmark studies on them, some context would be helpful for the reader here – maybe even as a short supplement or as a reference to the user manual. For example, how is the proposed approach conceptually different from existing tools like UCHIME etc? And why was it implemented as is? What was the (empirical?) motivation to do it like this, not otherwise? Personally, I am not very convinced of the performance of chimera-filtering algorithms overall and several recent pipelines side-step the issue more or less elegantly. In the case of NG-Tax (or other reference-based OTU callers), one could even argue that if the reference database is perfectly chimera-free, a closed-reference approach would not need a chimera filtering approach at all, or only one which is based on differential mapping of a sequence to two (highly unrelated) OTUs.
- Table 1 is very large and (on the PDF) unfortunately rotated by 90 degrees. I suggest to convert it into a supplemental Excel sheet which would be more reader-friendly.
- Figure 2 has rotated horizontal axis labels, a 90deg rotated legend – maybe that’s just due to formatting of the PDF. It is also difficult to read taxonomic names on the vertical axis in all-caps.
- “Consequently, these methods are more powerful than purely OTU-based methods, […].” While I agree with this sentence to a certain extent, I believe that the statement should be supported by referring to previous work on the topic. It is not necessarily consensus that 16S “sequence often correlates with phenotypic similarity in key features”, but it is even less clear to what extent phylogenetic diversity estimators capture this signal in a useful way. Arguably, a PD-estimator of UniFrac can only be as good as their underlying tree, which in turn is based on the (representative) sequences of OTUs and thus depends on many factors in the background.
- In particular, the weighted UniFrac measure used in this study seems to be more sensitive to quite a number of factors (including sequencing errors and inflation of small clusters, not irrelevant for the points made in this study) than its unweighted sister in my personal experience, and according to a number of researchers I have talked to on this point. However, since “personal experience” and “people I’ve talked to” are certainly not a dependable scientific source, and because performance on mock communities should not be severely impacted, I would formulate this as a suggestion and certainly not as a reviewer’s request: were the weighted UF-based results double-checked using unweighted UF and/or a non-phylogenetic method, such as Bray-Curtis?
- In the PCoA (Figure 5, A&C), it is quite hard to decide which method looks “better” purely based on visual impression, not least because the % variance explained on the axes is not equivalent. It would be good to see a more quantitative statement on which approach better recovers expected clusters from the mock communities. The most straightforward approach would be to perform MANOVA analyses, structured by the different factors to test for and then use the effect sizes to quantify the goodness of separation (or non-separation). I would suggest to run e.g. Anderson’s PERMANOVA ( http://www.entsoc.org/PDF/MUVE/6_NewMethod_MANOVA1_2.pdf; implementation available through the function “adonis” in the R package vegan) or ANOSIM to this end. Alternatively, samples could be clustered based on beta div and the resulting clusterings (or dendrograms) quantitatively compared to expectations based on different factors.
- Thank you for providing Supplementary Figures 1&2; they are informative in the interpretation of the presented data.
- Similarly, thank you for providing code and data as supplements!

I have read this submission. I believe that I have an appropriate level of expertise to confirm that it is of an acceptable scientific standard, however I have significant reservations, as outlined above.

## References

[1] TremblayJSinghKFernAKirtonESHeSWoykeTLeeJChenFDanglJLTringeSG: Primer and platform effects on 16S rRNA tag sequencing. *Front Microbiol*.2015;6: 10.3389/fmicb.2015.00771 771 10.3389/fmicb.2015.00771 26300854PMC4523815

[2] WestcottSLSchlossPD: De novo clustering methods outperform reference-based methods for assigning 16S rRNA gene sequences to operational taxonomic units. *PeerJ*.2015;3: 10.7717/peerj.1487 e1487 10.7717/peerj.1487 26664811PMC4675110

[3] KuninVEngelbrektsonAOchmanHHugenholtzP: Wrinkles in the rare biosphere: pyrosequencing errors can lead to artificial inflation of diversity estimates. *Environ Microbiol*.2010;12(1) : 10.1111/j.1462-2920.2009.02051.x 118-23 10.1111/j.1462-2920.2009.02051.x 19725865

[4] CallahanBJMcMurdiePJRosenMJHanAWJohnsonAJHolmesSP: DADA2: High-resolution sample inference from Illumina amplicon data. *Nat Methods*.2016;13(7) : 10.1038/nmeth.3869 581-3 10.1038/nmeth.3869 27214047PMC4927377

[5] AndersonM: A new method for non-parametric multivariate analysis of variance. *Austral Ecology*.2001;26(1) : 10.1111/j.1442-9993.2001.01070.pp.x 32-46 10.1111/j.1442-9993.2001.01070.pp.x

# Response

**Published:** 2018-11-14

**Authors:** Javier Ramiro-Garcia

**Affiliations:** University of Luxembourg, Luxembourg

**In their manuscript, the authors introduce NG-Tax, an open-source software for the (meta-)analysis of 16S rRNA-based microbiome datasets. Their tool focuses on an important and so-far arguably understudied aspect of microbial ecology research: the integration of results across studies, in view of both technical and biological variation.**

**The approach is interesting and addresses important points. In particular, several sequencing datasets of different mock communities were generated, even using different primer sets: this is great data to benchmark on, and many (most) other papers introducing tools do not provide benchmarks on such an array of real (mock) data. In general, I feel that this is very interesting work and that NG-Tax can be a promising alternative to existing tools in the field.**

We thank the reviewer for his nice comments and also his suggestions about the manuscript.

**However, there are several points that I feel would need to be addressed in order for the manuscript to stand tall, and for the reader to get a good understanding of how NG-Tax can be useful in practice.**

**Major comments: **

- **Even after reading the manuscript and online user manual repeatedly, I have to admit that it is not completely clear to me how NG-Tax works in detail, and in which points exactly it differs from existing approaches. Based on the introduction, I gather that NG-Tax relies on closed-reference OTU picking, but this is not mentioned explicitly anywhere in the text. Also, does reference-based OTU picking in NG-Tax rely on uclust? If yes, which version and parameters were used, and how do they differ from QIIME’s defaults? Also, the Background and Discussion sections do not elaborate on the various disadvantages of closed-reference approaches; most importantly, closed-ref only takes into account sequences matching the database and removes everything else. When integrating sequence data from different primer sets, this is arguably the most straightforward approach; however, the limitations should be discussed.**

Thanks for the suggestion. In the new version we included a more detailed Figure 1 including those unique aspects of NG-Tax.

We agree with the reviewer that close reference OTU picking has the disadvantage of only taking sequences into account that have a match in the database, and this is incompatible with having stable OTUs since databases change over time. For this reason, NG-Tax employs an open reference approach to remain independent of reference databases.

Different clustering algorithms also lead to different OTUs, and hence no clustering process is applied and the generation of OTUs is independent for each sample. Existing open reference approaches generate OTUs for the whole study by clustering the reads from all the samples together. Then, if new samples are included to a previous study, the OTUs need to be regenerated with the reads from the previous study and new samples together, which will lead to discrepancies in the former and new composition of the samples because some of the previous OTUs may not be present in the new analysis anymore.

Instead, in NG-Tax OTUs are generated sample by sample using the following strategy:

For each sample reads are ranked by read abundance and OTUs are added to an OTU table starting from the most abundant sequence until the read abundance is lower than a percentage defined by the user (recommended is at is 0.1%). Subsequently, the discarded reads are clustered to the OTU table allowing one mismatch.

In practical terms it is guided clustering where seeds are determined by abundance. The differences with an normal clustering approach is that there is no clustering to define the seeds, which allows seeds that differ as little as one nucleotide. The clustering is applied only afterwards to compensate for potential bias due to PCR and sequencing errors. Error is sequence-specific, and hence some sequences could be affected more than others. If a species specific amplicon is more prone to PCR or sequencing errors, the relative abundance of that particular OTU will be underestimated. But after clustering, OTUs more prone to error receive a higher percentage of discarded reads than others, this differential recovery helps to reestablish the true composition that was lost due to sequence specific error rates.

We substituted uclust by usearch in the scripts of the new version.

 

- **I gather from the text that NG-Tax’s main innovations are the use of primer-tailored reference databases and a different (more conservative) read abundance filtering scheme. It is perfectly valid to benchmark these against QIIME’s default settings; however, it would be great to see how QIIME performs with similarly conservative settings, to better understand where NG-Tax’s edge in performance comes from.**

We think that the main innovation of NG-Tax is the way OTUs are generated. This may seem counter-intuitive because it does not follow the standard approach but it is the discerning step compared with other existing pipelines. This innovative OTU generation algorithm is the reason of the NG-Tax’s edge in performance.

With QIIME those conservative thresholds cannot be used because the filtering percentage threshold is defined using the whole library and within a library there are samples that contain 20 times more reads than others. A conservative threshold like 0.1% is conservative for an average sample, not conservative for a big sample at all and extreme for small samples. Hence, OTUs present in only small samples can be discarded even if they represent 1% of that sample but less than 0.1% of the whole dataset. On the other hand, NG-Tax applies thresholds defined by sample accounting for sample heterogeneity.

In the manuscript we used the setting recommended by QIIME and described in Bokulich et al 2013 ^1^. For NG-Tax analysis we also employed recommended default settings so we thought that even if this is not optimal and has its limitations, this could be a fair approach. Nevertheless, we benchmarked with QIIME not to compare performances but rather to show that this dataset is not an exceptional case and the commonly reported problems such as many un- or poorly classified OTUs, inflated richness and diversity, taxonomic profiles that do not match the expected ones, region dependent taxonomic classification and results being highly dependent on minor changes in the experimental setup are also found in this dataset when standard approaches are used.

In Bokulich et al 2013, supplementary material 2; pages 8, 9 and 10, the authors compare expected composition against real sample composition using different parameters, one of them being OTU abundance, and the plot shows that the obtained profiles do not change much using different abundance thresholds.

Nonetheless, as suggested by the reviewer we reproduced the QIIME analysis with a 0.1% abundance threshold and this is included in the supplementary data. The results using 0.1% or 0.005% are consistent.

 

- **Regarding taxonomy assignments, it is valid to compare NG-Tax’s uclust-based approach to QIIME’s uclust-based approach. However, I believe that the gold standard continues to be the RDP Classifier, and it would be interesting to see a performance comparison to this tool (on the short-read data, not only on full-length reads). Also, how does taxonomic classification by NG-Tax differ conceptually from RTAX ( http://www.uio.no/english/services/it/research/hpc/abel/help/software/rtax.html)? I do believe that they are not equivalent, but the approaches appear somewhat related.**

In the manuscript we wanted to show that taxonomy assignment using short reads should be comparable with the assignment using the complete 16S rRNA gene (Figure 2). This is why we employed full length sequences. We could have included also RDP short read based taxonomy but the reads were too short for RDP, and hence genus and many times even family assignment could not be achieved with a minimum threshold value of 50%. In the supplementary data we supplied the theoretical compositions for all mock communities. The files for MC2 V4 and MC2 V5V6 contain all phylotypes and can be uploaded to the RDP classifier to verify the poor performance. In the new manuscript we substituted RDP for SILVA Incremental Aligner (SINA) to classify the full length sequences and we also updated the database in NG-Tax to SILVA 128, improving in both cases the classification.

I read the manuscript suggested by the reviewer and I can say that NG-Tax taxonomic classification is very similar to rtax.

The NG-Tax classifier works as follows:

For each OTU, usearch is used to retrieve hits for the forward and reverse reads against their respective trimmed reference database. Hits that are common between both reads are divided in 6 identity thresholds 100, 98, 97, 95, 92, 90. A hit belongs to a certain level, for example 97, when both reads have at least a 97 percentage identity with that hit. Using the highest available identity threshold, NG-Tax assigns the consensus taxonomy to the OTU if this taxonomy is supported for at least half of the hits. Genus, Family or Order remains unassigned if the maximum identity percentage level is lower or equal to 97%, 95% and 92% respectively. These are the main differences:

rtax clusters the reference database at 99%, while NG-Tax does not.

rtax averages the percentage identity for both reads and then considers the hits that have an averaged percentage identity 0.5% lower than the maximum averaged percentage identity as valid. NG-Tax does not average the percentage identities and uses fixed values 100, 98, 97, 95, 92 and 90 as thresholds.

For the rest they are indeed very similar approaches.

Therefore we have added rtax to the references and acknowledge in the manuscript that similar dynamic identity thresholds have been already employed to assign taxonomy. Furthermore, all the details about NG-Tax taxonomic assignment have been added to the user manual.

- **In general, the results on taxonomic classification are not discussed quantitatively. From Figures 3&4, the visual impression is that NG-Tax indeed better approximates expected taxonomic profiles than QIIME, but it is hard to quantify this from stacked bar charts. I would suggest to compute e.g. Euclidean or more sophisticated distances of classified taxonomic profiles to the expected distribution. Also, it would be interesting to see quantitative sensitivities and specificities (or F1-scores?) on the taxonomic assignments; particularly also when running on the exact same (more conservatively filtered) dataset for QIIME. Some numbers on specificity are provided in the Abstract and Conclusion sections – but I am not sure if specificity may be gained at the expense of sensitivity based on the more rigid read filtering upstream. **

As suggested by the reviewer, distances between compositional profiles and expected profiles are now shown in Figure 5. Distances between taxonomic profiles were calculated as the sum of the weighted differences. Given two taxonomical profiles x and y, for each taxa i, we defined the difference in abundance as difi(x,y)=( xi –yi) and a weighing factor wi as wi(x,y)=( xi –yi )/avg(xi + yi). Weighted difference was the result of multiplying the difference in abundance by its weighting factor. This weighting factor is useful to take into account the relative change and not only the absolute change, because a 1% absolute change becomes a 200% or 20% relative change depending on whether the expected abundance is 0.5% or 5% respectively. We performed t tests to compare the performance of NG-Tax versus QIIME from a quantitative point of view.

We have also included an Excel spreadsheet with compositional profiles in the supplementary data.

Figure 2 shows specificity of the taxonomical assignments and has been has been modified to improve readability.

The QIIME analysis at 0.1% abundance threshold can be found in the supplementary material.

- **As a suggestion, but certainly not as a request, I would recommend to maybe include additional, independent datasets to benchmark on. For example, Tremblay *et al. *(2015) have published data on mock communities sequenced with different primer sets and on different platforms. Such data could contribute to a yet more general assessment of NG-Tax performance.**

We thank the reviewer for the suggestion, but including new datasets will imply a rewrite of a big part of the manuscript. We consider that 49 samples can give an idea of NG-Tax performance. Additionally, we would like to mention that NG-Tax has been the reference method for 16S rRNA gene amplicon analysis in our lab for more than two years and has been used in more than 30 manuscripts that have been submitted or are in preparation. One of these manuscripts ^2^ was published before this manuscript. Since then another fifteen studies using NG-Tax have been published ^3-17^. These studies contain biological samples that belong to very different and specific environments and were sequenced both on MiSeq and HiSeq instruments. These will contribute to the assessment of NG-Tax performance, however these were not included in the current manuscript since they are accessible on the aforementioned publications.

**Minor comments (chronologically, not in order of importance): **

- **Background, “The consequence of this approach is that the ‘quality’ of the clustering of the reference set propagates to reference-picked OTUs.” I believe that as such, this statement is not fully valid or supported. In fact, the negative complement is arguably true: reference-based OTU picking against a “bad” reference can never provide “good” OTUs (a garbage-in, garbage-out problem, so to say). However, even with a good reference, a bad mapping algorithm can generate non-informative reference-based OTU sets. Schloss & Westcott have recently published a study which discusses this point, among others (Westcott & Schloss, 2015).**

With this sentence we did not imply that only ‘good quality’ is transferred from the clustered databases to the OTUs, we meant both, pros and cons are transferred. In fact, we agree that references have their limitations and clustered databases also contain bias due to clustering. For this reason NG-Tax employs a *de novo* OTU picking with no references or clustering involved.

- **Background, “However, in 16S rRNA gene amplicon sequencing every sequencing error could potentially lead to the false discovery of a new species.” I have two comments on this statement. First, I believe that the term “species” in this context can be misleading and I feel that the neutral term OTU or diversity unit would be more appropriate. Second, there is a large body of literature on how sequencing errors affect 16S-based diversity studies beyond the cited Bokulich et al paper (starting from Kunin *et al.*, 2010), and it would be worth to at least mention these, although an in-depth discussion would probably lead away from this study’s focus. Also, it may be worth mentioning recent algorithmic approaches to tackling this issue, such as DADA2 (Callahan *et al*., 2016).**

As suggested by the reviewer we rephrased the sequence to avoid the use of “species”. Now we stated: “However, in 16S rRNA gene amplicon sequencing every sequencing error could potentially lead to an incorrect OTU classification which may ultimately lead to the false discovery of a new phylotype”

We added Kunin et al 2010 and Callahan et al. 2016 to the references but we just wanted to point out that sequencing error is an important factor in 16S analysis rather than make an in-depth discussion about it.

- **Results & Discussion, chimera filtering. The implemented method for chimera filtering appears a little *ad hoc *and heuristic, although the proposed approach certainly makes sense intuitively. However, given the long history of “chimera-slaying” algorithms and the quite sobering benchmark studies on them, some context would be helpful for the reader here – maybe even as a short supplement or as a reference to the user manual. For example, how is the proposed approach conceptually different from existing tools like UCHIME etc? And why was it implemented as is? What was the (empirical?) motivation to do it like this, not otherwise? Personally, I am not very convinced of the performance of chimera-filtering algorithms overall and several recent pipelines side-step the issue more or less elegantly. In the case of NG-Tax (or other reference-based OTU callers), one could even argue that if the reference database is perfectly chimera-free, a closed-reference approach would not need a chimera filtering approach at all, or only one which is based on differential mapping of a sequence to two (highly unrelated) OTUs.**

First, we would like to recall that NG-Tax is not reference-based.

We fully agree with the reviewer opinion about ‘chimera-slaying’ algorithms. Chimera detectors are often validated using in-silico datasets generated by determining an initial set of valid sequences and a chimera formation pattern. This pattern or “rule” for chimera formation is commonly defined by considering that any two sequences in the initial dataset are equally probable to lead to a chimera and any nucleotide is equally probable to be the point in which these two sequences merge to form the chimera. It is conceivable that maybe the initial set is not representative of the sequences present in a specific real biological sample, not every pair of sequences has the same probability to form chimeras and not all the nucleotides may have the same odd to be the merging point of two sequences. Many different sequence sets can be selected as initial valid sequences and also many different chimera formation patterns can be chosen, but it is very difficult to really determine whether our choices mimic the way in which chimeras are formed in real sequencing data and therefore it is hard to verify if those in-silico created chimeras represent the chimeras that can be found in real sequencing samples. We consider that the proper validation should be using the real sequencing samples. If the chimera detection algorithm works, we would expect a very small number of non-assigned reads (since most chimeras should be aberrant). In case we have positive controls like MC, sequencing profiles and diversity should resemble the expected ones, and this is exactly what we observe with the results of NG-Tax.

We think that there are no perfect chimera-free databases, and a valid OTU can be found in the reference database and at the same time be a perfect combination of 2 other OTUs, especially for regions with lower variability (V4). If all those 3 OTUs are present in the same sample, how can we know whether it is a chimera or real?

In our opinion chimera detection is the weakest step in 16S pipelines because there is no satisfactory solution to the problem mentioned above. So the prevention against chimeras should come from the experimental design by reducing the PCR cycles and selecting regions of high variability. Chimera removal has many limitations and human supervision is recommended. For this reason, we decided to simplify the chimera detection as much as possible so the researcher can quickly identify why an OTU has been discarded. And also apply stringent parameters (100% identity) to avoid false positives. False negatives should be easier to detect afterwards since most of the chimeras should be aberrant.

In the manuscript of UCHIME they stated that “UCHIME searches for a chimeric alignment between a query sequence (Q) and two candidate parents (A and B)” and “candidate parents are required to have abundance at least λ times that of the query sequence, on the assumption that a chimera has undergone fewer rounds of amplification and will therefore be less abundant than its parents. The parameter λ is called the abundance skew, and by default λ=2 “, so NG-Tax approach is very similar to de novo UCHIME approach, but NG-Tax treats forward and reverse reads separately.

- **Table 1 is very large and (on the PDF) unfortunately rotated by 90 degrees. I suggest to convert it into a supplemental Excel sheet which would be more reader-friendly.**

As suggested by the reviewer Table 1 is now supplied as an excel spreadsheet in the supplementary material

- **Figure 2 has rotated horizontal axis labels, a 90deg rotated legend – maybe that’s just due to formatting of the PDF. It is also difficult to read taxonomic names on the vertical axis in all-caps.**

As suggested by the reviewer we modified Figure 2 to increase readability.

- **“Consequently, these methods are more powerful than purely OTU-based methods, […].” While I agree with this sentence to a certain extent, I believe that the statement should be supported by referring to previous work on the topic. It is not necessarily consensus that 16S “sequence often correlates with phenotypic similarity in key features”, but it is even less clear to what extent phylogenetic diversity estimators capture this signal in a useful way. Arguably, a PD-estimator of UniFrac can only be as good as their underlying tree, which in turn is based on the (representative) sequences of OTUs and thus depends on many factors in the background.**

Taxonomic assignment of the OTUs suffers from the same problems raised by the reviewer. Not always does 16S rRNA gene sequence similarity correlate with phenotypic similarity and the taxonomical assignment is as good as the reference database and the classifier employed. But having a composition barplot with OTUs named by number rather than by taxonomy would mean that all the information provided by the nucleotide sequence is discarded. This information may not be perfect but we cannot neglect that this information transformed into taxonomical assignment is useful at least to some extent.

The same criterion was applied to evaluate diversity. We used phylogenetic methods, which retain the information of the nucleotide sequence. We acknowledge the limitations but we argue that a sample containing 5 OTUs with a 99% pairwise sequence identity should not be given the same (potential) diversity that a sample containing 5 OTUs with less than 85% pairwise sequence identity. We consider that phylogenetic methods are more powerful because they use all information available, however, we should not over extrapolate the results. 16S rRNA gene amplicon sequencing should be taken as exploratory approach, whereas metagenomic and metatranscriptomic sequencing provides a more suitable and precise approach if we really want to focus on microbial functionality.

 

- **In particular, the weighted UniFrac measure used in this study seems to be more sensitive to quite a number of factors (including sequencing errors and inflation of small clusters, not irrelevant for the points made in this study) than its unweighted sister in my personal experience, and according to a number of researchers I have talked to on this point. However, since “personal experience” and “people I’ve talked to” are certainly not a dependable scientific source, and because performance on mock communities should not be severely impacted, I would formulate this as a suggestion and certainly not as a reviewer’s request: were the weighted UF-based results double-checked using unweighted UF and/or a non-phylogenetic method, such as Bray-Curtis?**

As suggested by the reviewer we have included the unweighted UniFrac and Bray-Curtis analysis in the supplementary material. The results obtained by all three methods are in concordance. 

- **In the PCoA (Figure 5, A&C), it is quite hard to decide which method looks “better” purely based on visual impression, not least because the % variance explained on the axes is not equivalent. It would be good to see a more quantitative statement on which approach better recovers expected clusters from the mock communities. The most straightforward approach would be to perform MANOVA analyses, structured by the different factors to test for and then use the effect sizes to quantify the goodness of separation (or non-separation). I would suggest to run e.g. Anderson’s PERMANOVA ( http://www.entsoc.org/PDF/MUVE/6_NewMethod_MANOVA1_2.pdf; implementation available through the function “adonis” in the R package vegan) or ANOSIM to this end. Alternatively, samples could be clustered based on beta div and the resulting clusterings (or dendrograms) quantitatively compared to expectations based on different factors.**

Thanks for the suggestion. In the new version of the manuscript we performed a more quantitative analysis of the sequencing data and the expected MC. We performed a permanova analysis under MC type factor and it was significant for both pipelines meaning that some of the variance is explained by the Mock type. But to really evaluate accuracy and reproducibility and compare pipelines performances we used pairwise distances and t tests (Figure 7 and Dataset 1).

 

- **Thank you for providing Supplementary Figures 1&2; they are informative in the interpretation of the presented data.**

Thank you.

- **Similarly, thank you for providing code and data as supplements!**

Thank you.

References:

1. Bokulich NA, Subramanian S, Faith JJ, et al. Quality-filtering vastly improves diversity estimates from Illumina amplicon sequencing. Nat Methods 2013;10:57-9.

2. Timmers PH, Widjaja-Greefkes HC, Ramiro-Garcia J, et al. Growth and activity of ANME clades with different sulfate and sulfide concentrations in the presence of methane. Front Microbiol 2015;6:988.

3. Giatsis C, Sipkema D, Ramiro-Garcia J, et al. Probiotic legacy effects on gut microbial assembly in tilapia larvae. Sci Rep 2016;6:33965.

4. Atashgahi S, Lu Y, Ramiro-Garcia J, et al. Geochemical Parameters and Reductive Dechlorination Determine Aerobic Cometabolic vs Aerobic Metabolic Vinyl Chloride Biodegradation at Oxic/Anoxic Interface of Hyporheic Zones. Environ Sci Technol 2017;51:1626-1634.

5. Atashgahi S, Lu Y, Zheng Y, et al. Geochemical and microbial community determinants of reductive dechlorination at a site biostimulated with glycerol. Environ Microbiol 2017;19:968-981.

6. Azman S, Khadem AF, Plugge CM, et al. Effect of humic acid on anaerobic digestion of cellulose and xylan in completely stirred tank reactors: inhibitory effect, mitigation of the inhibition and the dynamics of the microbial communities. Appl Microbiol Biotechnol 2017;101:889-901.

7. Dieho K, van den Bogert B, Henderson G, et al. Changes in rumen microbiota composition and in situ degradation kinetics during the dry period and early lactation as affected by rate of increase of concentrate allowance. J Dairy Sci 2017;100:2695-2710.

8. Lu Y, Ramiro-Garcia J, Vandermeeren P, et al. Dechlorination of three tetrachlorobenzene isomers by contaminated harbor sludge-derived enrichment cultures follows thermodynamically favorable reactions. Appl Microbiol Biotechnol 2017;101:2589-2601.

9. van Lingen HJ, Edwards JE, Vaidya JD, et al. Diurnal Dynamics of Gaseous and Dissolved Metabolites and Microbiota Composition in the Bovine Rumen. Front Microbiol 2017;8:425.

10. Paulo LM, Ramiro-Garcia J, van Mourik S, et al. Effect of Nickel and Cobalt on Methanogenic Enrichment Cultures and Role of Biogenic Sulfide in Metal Toxicity Attenuation. Front Microbiol 2017;8:1341.

11. van Gastelen S, Visker M, Edwards JE, et al. Linseed oil and DGAT1 K232A polymorphism: Effects on methane emission, energy and nitrogen metabolism, lactation performance, ruminal fermentation, and rumen microbial composition of Holstein-Friesian cows. J Dairy Sci 2017;100:8939-8957.

12. Gerritsen J, Hornung B, Renckens B, et al. Genomic and functional analysis of Romboutsia ilealis CRIBT reveals adaptation to the small intestine. PeerJ 2017;5:e3698.

13. van der Waals MJ, Pijls C, Sinke AJC, et al. Anaerobic degradation of a mixture of MtBE, EtBE, TBA, and benzene under different redox conditions. Appl Microbiol Biotechnol 2018;102:3387-3397.

14. Umanets A, de Winter I, F IJ, et al. Occupancy strongly influences faecal microbial composition of wild lemurs. FEMS Microbiol Ecol 2018;94.

15. Steinert G, Gutleben J, Atikana A, et al. Coexistence of poribacterial phylotypes among geographically widespread and phylogenetically divergent sponge hosts. Environ Microbiol Rep 2018;10:80-91.

16. Gu F, Borewicz K, Richter B, et al. In Vitro Fermentation Behavior of Isomalto/Malto-Polysaccharides Using Human Fecal Inoculum Indicates Prebiotic Potential. Mol Nutr Food Res 2018;62:e1800232.

17. Dat TTH, Steinert G, Thi Kim Cuc N, et al. Archaeal and bacterial diversity and community composition from 18 phylogenetically divergent sponge species in Vietnam. PeerJ 2018;6:e4970.
